# *Hoxa5* Activity Across the Lateral Somitic Frontier Regulates Development of the Mouse Sternum

**DOI:** 10.3389/fcell.2022.806545

**Published:** 2022-04-26

**Authors:** Kira Mitchel, Jenna M. Bergmann, Ava E. Brent, Tova M. Finkelstein, Kyra A. Schindler, Miriam A. Holzman, Lucie Jeannotte, Jennifer H. Mansfield

**Affiliations:** ^1^ Department of Biology, Barnard College, Columbia University, New York, NY, United States; ^2^ Department of Molecular Biology, Medical Biochemistry and Pathology, Faculty of Medicine, Université Laval, Québec, QC, Canada; ^3^ Centre de Recherche sur le Cancer de l'Université Laval, CRCHU de Québec‐Université, Laval (Oncology Axis), Québec, QC, Canada

**Keywords:** *Hoxa5*, sternum, somites, lateral plate mesoderm, lateral somitic frontier, skeletal patterning

## Abstract

The skeletal system derives from multiple embryonic sources whose derivatives must develop in coordination to produce an integrated whole. In particular, interactions across the lateral somitic frontier, where derivatives of the somites and lateral plate mesoderm come into contact, are important for proper development. Many questions remain about genetic control of this coordination, and embryological information is incomplete for some structures that incorporate the frontier, including the sternum. Hox genes act in both tissues as regulators of skeletal pattern. Here, we used conditional deletion to characterize the tissue-specific contributions of *Hoxa5* to skeletal patterning. We found that most aspects of the *Hoxa5* skeletal phenotype are attributable to its activity in one or the other tissue, indicating largely additive roles. However, multiple roles are identified at the junction of the T1 ribs and the anterior portion of the sternum, or presternum. The embryology of the presternum has not been well described in mouse. We present a model for presternum development, and show that it arises from multiple, paired LPM-derived primordia. We show evidence that HOXA5 expression marks the embryonic precursor of a recently identified lateral presternum structure that is variably present in therians.

## Introduction

The post-cranial musculoskeletal system derives primarily from two embryonic sources: the somites and the lateral plate mesoderm (LPM). These mesodermal populations have distinct developmental programs, genetic control, and evolutionary histories, yet they must develop in coordination to permit the functional integration of their derivatives. Somites and LPM each give rise to skeletal tissue, including cartilage and bone, and to connective tissue such as tendons, ligaments, and muscle connective tissue (reviewed in (Christ et al., 2007; Prummel et al., 2020)). In contrast, all skeletal muscle is somite-derived, with the exception of some cranial muscles (reviewed in (Yahya et al., 2020)).

Musculoskeletal structures can be categorized as primaxial or abaxial depending on the source of their connective tissues (Burke & Nowicki, 2003). In primaxial structures, which include the vertebral column and proximal ribs, all musculoskeletal tissues are entirely somite-derived. Abaxial structures include the limbs, most of the limb girdles (shoulder and pelvis), the sternum, and in mice, the distal T1 rib (Durland et al., 2008). In these structures, all connective and most skeletal tissue is LPM-derived, but muscles arise from progenitors that migrate ventrally from the somites into LPM territory. In some abaxial structures, such as the distal T1 rib, the cartilage also arises from ventrally migrating somite-derived cells. The border between the primaxial and abaxial domains was first mapped in chick and has been termed the lateral somitic frontier (LSF (Nowicki & Burke, 2000)). The LSF has also been mapped in mouse embryos using the *Prx1-Cre* transgene (Durland et al., 2008). A few structures, described as incorporating the frontier, have both primaxial and abaxial portions. In mice, this includes the scapula (whose dorso-medial border is somite-derived), the intercostal muscles, and the junction of the ribs and sternum (described further below) (Durland et al., 2008).

The primaxial/abaxial distinction has facilitated understanding of developmental phenotypes, which often differ in or are confined to derivatives of one domain or another, suggesting that these are at least partially independent developmental fields (Burke & Nowicki, 2003). It is also consistent with an instructive role for connective tissue in musculoskeletal patterning, growth and homeostasis (reviewed in (Sefton & Kardon, 2019)). Finally, the primaxial/abaxial distinction is important for interpreting patterns and potential constraint of morphological evolution due to required interaction between the two tissues (reviewed in (Shearman & Burke, 2009)).

Indeed, somites and LPM have distinct evolutionary histories (Liem et al., 2001). For example, somites arose in basal (invertebrate) chordates and likely gave rise to the axial system of muscles and the connective tissues that attached them to the body wall (laterally) and notochord (medially), permitting locomotion (reviewed in Willey, 1894). Somites also gave rise to the earliest-evolved post-cranial skeleton: the vertebral column. Paired appendages evolved later, with skeletal tissue developing instead from LPM, but with somite-derived muscle migrating to populate them (reviewed in (Tanaka & Onimaru, 2012)). In tetrapods, the elaboration and diversification of limbs and limb girdles expanded the contribution of LPM to musculoskeletal development, and would have necessitated novel interactions across the lateral somitic frontier to preserve an integrated musculoskeletal system.

One region where such integration must occur is at the junction of the sternum and the ribs. The sternum evolved in tetrapods as an extension of the pectoral girdle. It functions as an attachment site for pectoral muscles that facilitate the transfer of body weight to the forelimbs. Rib-sternum articulation evolved secondarily in some tetrapods, serving to strengthen the ribcage, and has also been adapted for respiratory function. Indeed, sternal anatomy varies across tetrapod groups and reflects diverse modes of locomotion and respiration (Liem et al., 2001; Scaal, 2021). In most living mammals, the sternum contains three segments: the anterior sternum, or presternum (sometimes called the manubrium), the mesosternum and the xiphoid process. The presternum articulates anteriorly with the clavicles (in species where clavicles are present) and posteriorly with the second pair of ribs. The first pair of ribs joins the presternum laterally and this attachment is morphologically and functionally unique among rib attachments. The mesosternum extends from the second rib to the last attached (true) rib and is segmented in most mammals, made up of ossified sternebrae alternating with cartilaginous joints at the points of rib attachment; these provide flexibility for diaphragm-based respiration. The xiphoid process is a thickened plate at the posterior end the sternum that serves as an attachment point for diaphragm muscles.

The mesosternum develops from paired structures called sternal bars, and their embryology has been well-described in classical studies of mammals and birds (for example, (Gladstone & Wakeley, 1932; Fell, 1939; Chen, 1952a, 1952b, 1953; Chevallier, 1975). The sternal bars arise in the axillary region and derive from the *Tbx5*-expressing forelimb field (Gladstone & Wakeley, 1932; Chen, 1952a; Bickley & Logan, 2014). They migrate to the midline of the ventral body wall, where they fuse to form the sternum and displace existing mesenchyme, some of which undergoes cell death (Chen, 1952a). Rib anlagen, which grow ventrally from the somites into the body wall, fuse with the sternal bars during their migration. Sternal bars subsequently provide force that “pulls” the rib primordia toward the midline (Chen, 1952b), and conditional deletion of *Tbx5* in mouse LPM results in a complete loss of the sternal bars, and secondary failure of the ribcage to close ventrally (Bickley & Logan, 2014). The segmented structure of the mammalian sternum arises secondarily because the rib primordia inhibit sternum ossification at their attachment points via an unknown signal (Chen, 1953).

The embryology of the presternum has been less studied. However, there is evidence that in mammals it arises from the fusion of multiple embryonic cartilage condensations (Gladstone & Wakeley, 1932; Rodríguez-Vázquez et al., 2013; Buchholtz et al., 2020). These include the anterior sternal bars and an additional midline condensation proposed homologous to the interclavicle, which is an unpaired midline bone present in most synapsids but that is not present as a separate skeletal structure in therian mammals. The presence of an additional paired lateral element at the position of rib 1 articulation was proposed by a comparative study of presternum anatomy from extant and fossil mammals and from medical CT scans showing variably present lateral skeletal structures in human presternae (Buchholtz et al., 2020). Lineage tracing confirmed the LPM origin of the R1 attachment site (Durland et al., 2008) and a neural crest contribution has also been reported in the anterior sternum (Matsuoka et al., 2005). However, a thorough description of presternum development in a mammalian model system has been lacking. Thus, the number and origin of its primordia, as well as their embryological tissue of origin and relationship to the lateral somitic frontier, is unknown, as are potential signals acting across the frontier to coordinate rib-sternum interactions. Hox genes are a good candidate for playing a role in this latter activity.

Hox genes globally pattern anterior-posterior fates, including in both somite and LPM derivatives, and loss-of-function studies have demonstrated patterning roles in both primaxial and abaxial structures (reviewed in (Mallo et al., 2010)). Vertebrate Hox genes are expressed in a nested, colinear pattern in somites, but their expression boundaries are less regular with respect to cluster organization in the LPM, and often differ from those in somites (Burke et al., 1995). Heterotopic transplantation of presomitic mesoderm reveals that both segmental identity and Hox expression become determined prior to somite segmentation; primaxial structures develop according to their original location and maintain their own Hox code after transplantation. In contrast, the segmental identity of abaxial structures is governed by Hox expression in the LPM: muscle and skeletal progenitors that migrate across the lateral somitic frontier following heterotopic transplantation adopt the morphology and Hox code associated with the surrounding LPM (Kieny et al., 1972; Murakami & Nakamura, 1991; Nowicki & Burke, 2000).

In order to better understand how developmental programs are coordinated across the lateral somitic frontier, and specifically at the point of forelimb attachment, we took two approaches. In the first, we examined the tissue-specific requirements for *Hoxa5* in somites vs. LPM for mouse skeletal development. *Hoxa5* is a good model because of its non-redundant skeletal phenotypes affecting forelimb attachments and vertebral segments spanning the cervical-thoracic transition (reviewed in (Jeannotte et al., 2016)). Our results showed that *Hoxa5* null-associated skeletal phenotypes can be reproduced by tissue-specific *Hoxa5* deletion in somites or LPM, identifying the tissues in which it is required. Interestingly, LPM-specific deletion produced a novel phenotype, suggesting that coordinated HOXA5 expression across the frontier may be necessary for some aspects of its role in presternum development. In a second approach, and to better contextualize these genetic results, we characterized mouse presternum development at a series of stages with reference to the cartilage condensations, tissues of origin, and the relative role of *Hoxa5.*

Together, our results confirm and extend previous findings about the origin of the presternum as a composite structure. We show that the mouse presternum, including what is commonly referred to as the manubrium, is composed of at least five independent, paired mesenchymal condensations, all of which are primarily LPM-derived and lack contribution from somites. Molecular subdivision of one presternal element is provided by the differential expression of HOXA5 specifically at the points of rib 1 attachment, and this same region is disrupted in *Hoxa5* mutants. Thus, we propose that HOXA5 expression molecularly marks a previously-identified lateral element of the therian presternum. Together, our results shed light on the development of a structure arising at the lateral somitic frontier, and provide a genetic dissection of *Hox* activity spanning this junction.

## Results

### Distinct Phenotypes Result From Tissue-Specific Deletion of *Hoxa5* in Somites or LPM

**FIGURE 1:**
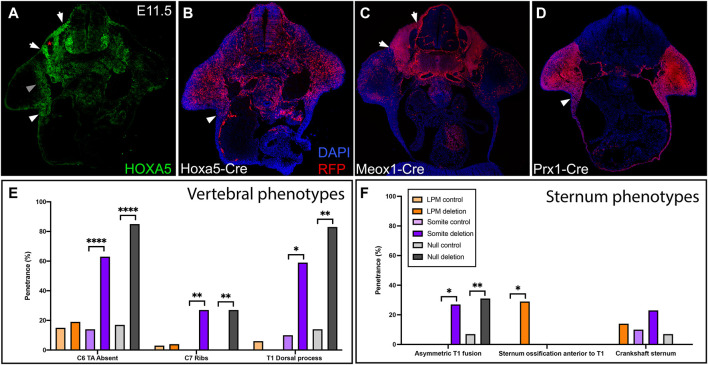
*Hoxa5* expression in somites and LPM. **(A)** HOXA5 protein expression and **(B)** domain of cells with an expression history of *Hoxa5* at E11.5, prior to migration of axial skeletal progenitors across the lateral somatic frontier. Compare to the location of cells derived from **(C)** somites, labelled with Meox1-Cre, and **(D)** LPM, labelled with Prx1-Cre. White arrows mark the lateral (left) and medial (right) borders of the somites. White arrowheads indicate the axillary region known to contain sternal bar progenitors. Grey arrowhead indicates additional HOXA5 expression in the limb bud, and asterisk marks the myotome (see text). **(E,F)** Summary of skeletal phenotypes following conditional *Hoxa5* deletion. Frequency of *Hoxa5* associated phenotypes in the **(E)** C6-T1 vertebrae and **(F)** sternum at E18.5. *p < 0.05, **p < 0.01, ****p < 0.0001, Fisher’s exact test. Complete genotypes of control and experimental groups are given in Supplementary Table S1.

HOXA5 protein is expressed in both somites and LPM, in adjacent domains that flank the lateral somitic frontier. This can be observed in a brachial somite at E11.5, just prior to the migration of rib and axial muscle progenitors, by comparison of HOXA5 expression and Cre-based lineage label (Figures 1A,B) to position-matched sections from embryos lineage-labeled for somites and LPM (Figures 1C,D) using Meox1-Cre (Jukkola et al., 2005) and Prx1-Cre (Logan et al., 2002), respectively. A comparison of these latter two images illustrates the position of the lateral somitic frontier. As previously described (Holzman et al., 2018), HOXA5 is expressed broadly in somites (between the two arrows, Figures 1A,C), with the exception of prospective skeletal muscle (asterisk and (Holzman et al., 2018)). In LPM, expression is observed in the limb field, including mesenchyme of the proximal forelimb (grey arrowhead) and the axillary region (white arrowheads in Figures 1A,B,D) where the sternal progenitors are found (Bickley & Logan, 2014). In contrast, few HOXA5-expressing cells are found in the LPM-derived ventral body wall mesenchyme. Similar to HOXA5 protein, Hoxa5-Cre activity visualized with an RFP reporter reveals a similar but somewhat broader spatial domain of cells with a *Hoxa5* expression history (especially in the forelimb) reflecting the dynamic nature of *Hoxa5* expression (Figure 1B; Bérubé-Simard & Jeannotte, 2014; Holzman et al., 2018). Together, these expression data raise the question of which domain(s) of *Hoxa5* activity mediate skeletal patterning, and whether somite and LPM activity is functionally independent for skeletal structures that incorporate the frontier.

To test these questions, we deleted *Hoxa5* in somites or in LPM with the *Hoxa5* ^*flox*^ conditional allele (Tabariès et al., 2007) and tissue-specific Cre lines. Skeletal phenotypes were examined at E18.5, with a focus on the sixth cervical to first thoracic (C6-T1) segments and on the sternum: regions that span the cervical-thoracic transition, that contain a mixture of primaxial, abaxial, and transitional strucutres, and that also include the most penetrant *Hoxa5* phenotypes (Jeannotte et al., 1993). Results are summarized in Figures 1E,F and detailed in Supplementary Table S1. To account for possible effects of genetic background introduced from the Cre lines, we included data for littermate controls from each cross.

### Homeotic Transformations Involve Somitic *Hoxa5* Activity

**FIGURE 2:**
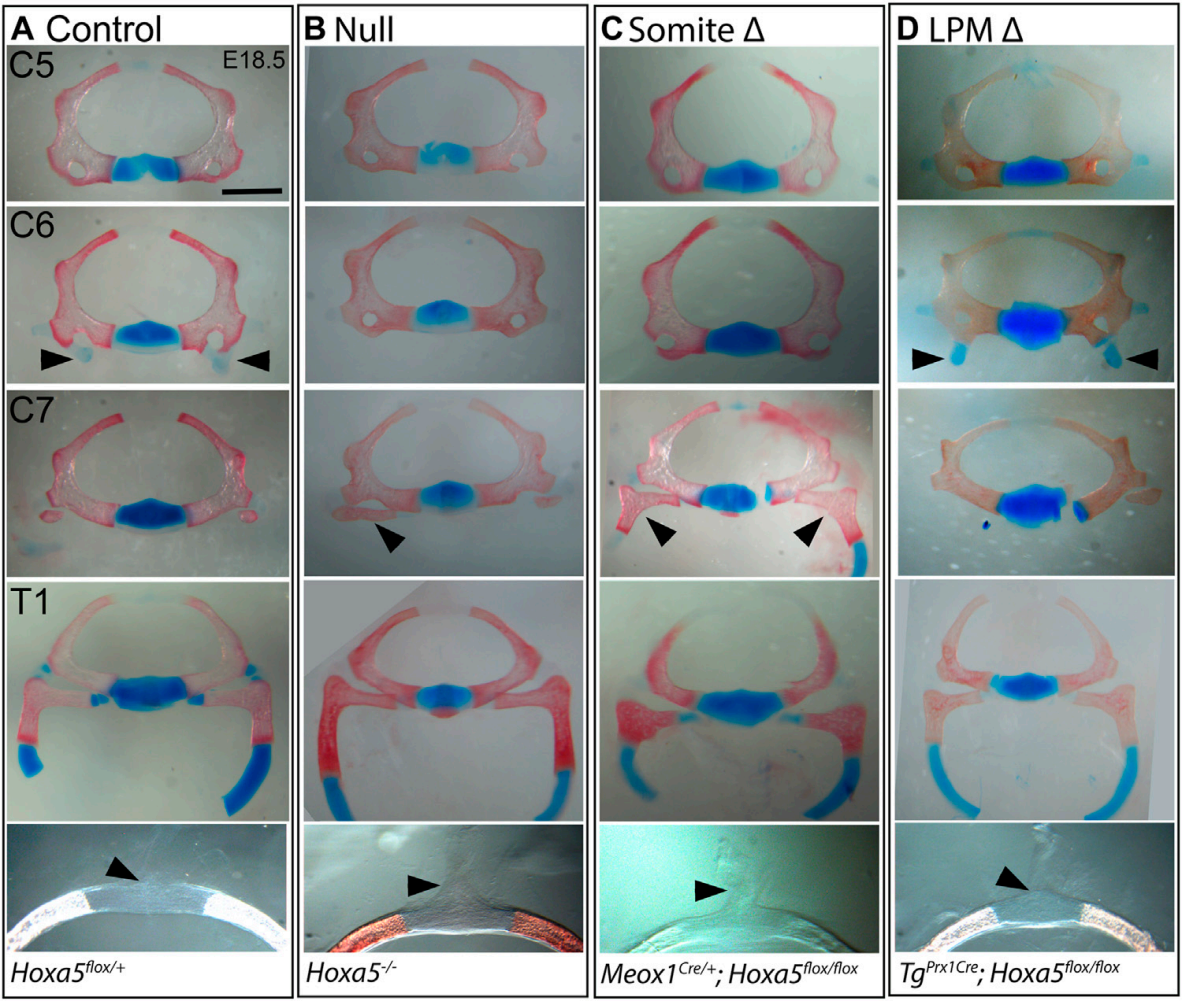
Vertebral phenotypes associated with conditional *Hoxa5* deletion at E18.5. Alcian blue and Alizarin red staining of vertebrae from control **(A)**, null **(B)**, somite deleted **(C)**, and LPM-deleted **(D)** embryos. Arrowheads indicate tuberculum anterior (TA) on C6, ribs on C7 and the position of the dorsal process on T1 (absence of a dorsal process indicated by a tilted arrowhead). Note that the T1 ribs have been cut within the cartilaginous, distal portion for photography. Scale bar 1 mm (top four rows) or 0.47 mm (bottom row).

The vertebrae are somite-derived and, except for the distal T1 rib, are entirely primaxial (Durland et al., 2008). Consistent with this, all vertebral phenotypes previously associated with *Hoxa5* loss-of-function (Jeannotte et al., 1993) were recapitulated by conditional deletion of *Hoxa5* in somites with Meox1-Cre (Figures 1E, 2). These included loss of the tuberculum anterior on C6, which is considered an anterior transformation of C6 to C5. Ectopic C7 ribs were also observed, interpreted as a posterior transformation of C7 to T1. As was previously reported for the null allele, C7 ribs could be unilateral or bilateral and varied in extent and in whether they were free or fused to the T1 rib or sternum (shown below). Cartilage nodules were frequent in all genotypes and were not considered ribs (see control embryo in Figure 2A and not shown). Finally, somite-specific *Hoxa5* deletion led to ectopic formation of dorsal processes on T1, considered a posterior transformation of T1 to T2 (Figures 1E, 2C). Note that at E18.5 the dorsal process was not yet positive for Alcian blue, but mesenchymal condensations could be observed. In contrast, none of these vertebral phenotypes were observed following LPM-specific *Hoxa5* deletion with Prx1-Cre (Figures 1F, 2D). These results indicate that, as expected, somite-specific *Hoxa5* activity is responsible for patterning of primaxial skeletal structures.

### Rib-Sternum Attachment Phenotypes Involve Somitic *Hoxa5* Activity

In addition to homeotic transformations, *Hoxa5* null mutants present rib fusions, bifurcations and asymmetric sternal attachment involving the first (T1) ribs and in some cases ectopic C7 ribs (Jeannotte et al., 1993). These phenotypes are consistent with a defect in rib guidance during segmental outgrowth across the lateral somitic frontier, and/or with altered recognition between T1 and a defined location on the presternum. Interestingly, these rib defects were all reproduced by conditional deletion of *Hoxa5* in somites but not by deletion of *Hoxa5* in LPM.

**FIGURE 3:**
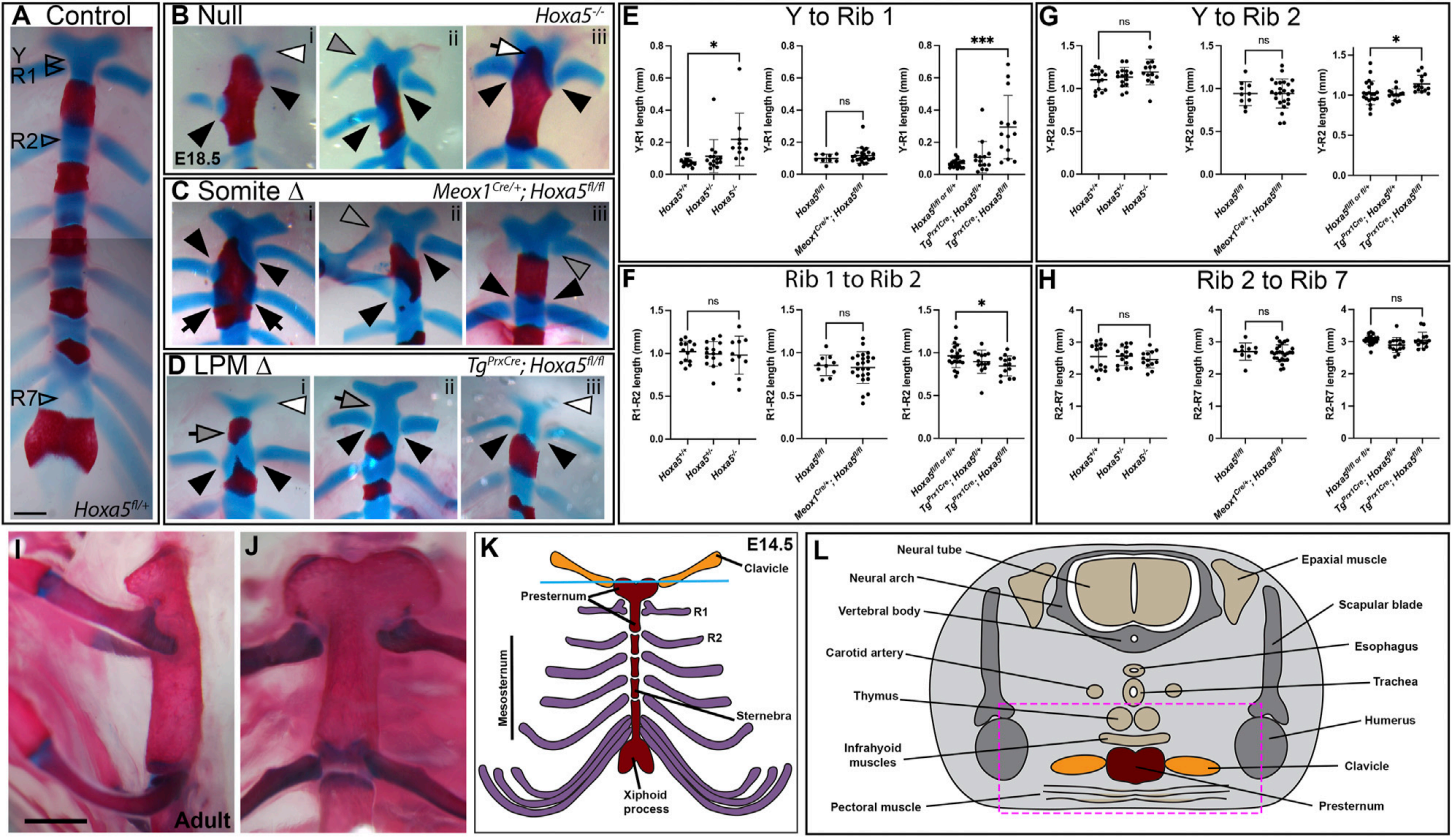
Sternum phenotypes associated with conditional *Hoxa5* deletion at E18.5. **(A)** Control sternum at E18.5. Arrowheads indicate the positions at which measurements were taken for data shown in panels **(E**–**H)**. **(B**–**D)** Examples of anterior sternum phenotypes. In all panels, filled black arrowheads indicate T1 ribs, black arrows indicate T2 ribs, and grey arrowheads indicate C7 ribs. White arrowheads indicate Y-shaped cartilage reduction, and white arrow in **(Biii)** indicates ectopic ossification of the Y-shaped cartilage. Grey arrows in **(Di,ii)** indicate an elongated sternum region between the Y-shaped cartilage and T1 attachment position, which was sometimes associated with an ectopic ossification. Scale bar: 500 µm. **(E**–**H)** Comparative lengths of sternal regions between arroweads indicated in **(A)**. In each panel, null crosses are graphed on the left, somite-specific deletion in the middle and LPM-specific deletion on the right. Bars and brackets indicate the mean and standard deviation, respectively. *p < 0.05, **p < 0.01, ***p < 0.001, n.s. not significant, Welch’s *t*-test. **(I,J)** Adult sternum with T1 and T2 attachments shown in side **(I)** or front **(J)** views. **(K)** Schematic of E14.5 sternum and ribs attachments. **(L)** Schematic of a cross-sections through an E14.5 embryo at the axial level indicated by the blue line in **(K)**.

In wild-type embryos, the T1 ribs fuse symmetrically and at a consistent position on the presternum, at the base of the Y-shaped cartilage (Figure 3A). Rib attachment defects are shown in Figure 3 and Supplementary Figure S1. Asymmetric attachment of T1 ribs to the sternum was frequent in both null embryos and following somite-specific deletion (Figures 3B,C, i,ii black arrowheads indicate T1 ribs in all panels). A T1 bifurcation following conditional deletion of *Hoxa5* in somites is shown in Supplementary Figure S1Civ. When they formed, C7 ribs were sometimes free (Supplementary Figure S1, compare **A** to **Bi,Ci**), but others fused with the T1 rib (Supplementary Figure S1Cii), or were complete ribs, fused to the sternum in the position normally occupied by T1 (Supplementary Figure S1Ciii, grey arrowheads indicate C7 ribs in all panels). All of these phenotypes were previously described for *Hoxa5* null embryos (Jeannotte et al., 1993). In contrast, no rib asymmetries, fusions or bifurcations were observed following *Hoxa5* conditional deletion in LPM.

We hypothesized that asymmetric T1 fusion could be caused by physical displacement of one rib due to presence an extra cervical rib on the same side. This was not the case, however, because there was no correlation between aberrant T1 rib fusion and an ipsilateral C7 rib. Of embryos with asymmetric T1 rib fusions, 2/4 null embryos and 2/7 following somite-specific *Hoxa5* deletion lacked an ectopic C7 rib on the same side (although we cannot rule out presence of ectopic intercostal muscle or connective tissue). Of those that did have ipsilateral C7 ribs, some were free, others fused to T1, and others fused to the sternum. Thus, the symmetric outgrowth and fusion of T1 ribs is apparently an independent phenotype from the presence of C7 ribs, and consistent with hypothesized loss of positional information either during rib migration and/or during presternum fusion. In one embryo with somite-specific deletion, in which a C7 rib was fused to the sternum, both T1 ribs shifted their attachment symmetrically to the normal T2 position (Figure 3Ciii; Supplementary Figure S1Ciii). This phenotype has been previously described in null embryos (Jeannotte et al., 1993) but was not observed in our null samples.

The posterior fusion of T1 ribs, particularly in the latter example, could be interpreted as a homeotic transformation of T1 to T2. This did not appear to be the case, however, based on sternum morphology. In wild-type mice (and other mammals), the mesosternum segments at the positions rib attachment (T2–T7), but not at the T1 attachment (Figures 3I,J). At E18.5, the future mesosternal joints are visible as thin discontinuities in Alcian blue staining, which are never observed at T1. Following somite-specific *Hoxa5* deletion, ectopic mesosternum-like segmentation was not observed at the position of T1 fusion, even when T1 ribs fused at the T2 position (not shown). This indicates that the joint maintained at least this aspect of T1 identity and was not transformed to a mesosternal-type joint. The T1 distal ribs are also morphologically distinct from all other ribs in adults because they make a double-contact with the sternum (Figures 3I,J, and see below). However, this morphology has not developed at E18.5 and thus could not be scored with Alcian blue staining.

Finally, it was previously reported that the *Hoxa5* skeletal phenotype involves asymmetric fusion of all or most ribs, resulting in bifurcated or fused sternebrae, also called a crankshaft sternum (Jeannotte et al., 1993). This phenotype has been hypothesized to result from asymmetric fusion of the ribs to the sternal bars, or from asymmetric fusion of the sternal bars. This in turn subsequently disrupts the regular alternation of ossified sternebrae and cartilaginous joints because the ribs are known to inhibit sternum ossification at points of contact (Chen, 1953), and likely does not reflect a patterning change in the sternum itself. We observed the crankshaft sternum phenotype at low frequency in both conditional knockout crosses, but occurrence was statistically indistinguishable from controls (Figure 1F; Supplementary Figure S1Cv).

### A Novel Presternum Phenotype Involves *Hoxa5* Activity in LPM

A novel phenotype not described in *Hoxa5* null embryos was produced by LPM-specific deletion of *Hoxa5* with Prx1-Cre: an additional ossification formed within the presternum, anterior to the T1 attachment (Figures 1F, 3Di; Supplementary Figure S1D, grey arrows). Further examination revealed that some embryos showed presternum elongation even when no extra ossification was present (Figure 3Dii, grey arrow). We therefore measured absolute and relative lengths of different sternal regions in all samples (measurement positions marked in Figure 3A). This confirmed that the distance from the base of the Y-shaped cartilage to the center of the T1 attachment was significantly increased following LPM-specific deletion (Figure 3E). Conversely, the distance between the T1 and T2 attachments, which is typically greater than that between mesosternum rib pairs, was significantly reduced (Figure 3F). However, the overall distance between Y and T2 was significantly increased (Figure 3G) confirming an overall elongation of the presternum. In contrast, the length of the mesosternum, measured between the T2 and T7 attachments, was not affected (Figure 3H). Similar to the case above, the extra sternebra above the T1 attachment might indicate a posterior shift in identity. However, as in the examples above, even when an extra ossification was present anterior to the T1 attachment, the sternum retained a T1-like morphology because it did not segment to form a joint (not shown).

Sternum lengths were also measured following somite-specific or complete *Hoxa5* deletion (here, specimens with asymmetric T1 fusion were excluded from measurements involving T1 position but included in Y-T2 and T2–T7 measurements). No significant differences in the lengths of sternal regions were observed following somite-specific deletion of *Hoxa5* (Figures 3E–H)*.* However, the Y to T1 length was significantly increased in the null embryos, even excluding those with T1 asymmetric fusion. However, the elongation was less severe than following LPM-specific deletion and never involved an ectopic ossification anterior to T1 (Figure 3E).

Together, these results show that *Hoxa5* activity specific to the LPM is responsible for the presternum phenotypes. The observation that the elongation of the presternum is more severe following LPM-specific deletion of *Hoxa5* compared to complete loss-of-function implies that *Hoxa5* activities in somites and LPM are not completely independent. Rather, this could indicate that they act coordinately to pattern the presternum; if so, a mismatch in positional information following conditional deletion could be expected to produce such a novel phenotype relative to complete deletion. This possibility is discussed further below.

Finally, the Y-shaped cartilage was often reduced following either LPM-specific deletion or in null embryos (Figures 3Bi,Di,iii, white arrowheads) but not following somite-specific deletion.

### Sternum Ossification Phenotypes are Most Prevalent in *Hoxa5* Null Embryos

In wildtype embryos, rib primordia inhibit ossification of the sternum at contact points (Chen 1953). In E18.5 embryos, the sternum anterior to T1 is completely cartilaginous (Figure 3A) although in adults this region does ossify (Figures 3I,J). In *Hoxa5* null embryos, early sternal ossification occurred frequently at points of T1 rib contact (12/26 T1 ribs; for example, Figures 3Bi,iii). Ectopic ossification often extended anteriorly into the Y-shaped cartilage (7/13 null embryos; Figure 3Biii and Supplementary Figure S1Bii). This phenotype was observed in one embryo following somite-specific deletion (Figure 3Ci) and involved one T1 and both T2 ribs, but never following LPM-specific deletion. Together, this implicates *Hoxa5* in negatively regulating ossification of the presternum, but does not clearly identify the location of its activity in this role.

### Embryonic Development of the Mouse Presternum and the Role of *Hoxa5*

The results above showed that *Hoxa5* plays distinct and largely independent roles in the somites and LPM. However, a novel phenotype following LPM conditional deletion indicates that tissue-specific roles may not be purely additive in the presternum. Overall, the function of *Hoxa5* in presternum development could be better understood if described in reference to presternum embryology, which is incompletely characterized.

### Presternum Development is Disrupted by *Hoxa5* Loss of Function

In an adult skeletal specimen, the unique nature of the anterior sternum and T1 articulation is apparent, including the absence of sternal segmentation at the T1 joint and the morphology of the distal T1 ribs, which form a double attachmentto the sternum, unlike the mesosternal ribs. (Figures 3I,J, and diagrammed in Figure 3K).

A recent description in human embryos suggests that multiple cartilage condensations contribute to the presternum (Rodríguez-Vázquez et al., 2013). This study showed that interclavicular (IC) mesenchyme arises between and continuous with the clavicle condensations and was proposed to have neural crest origin, similar to the endochondral portion of the clavicle (Matsuoka et al., 2005). IC mesenchyme later expands caudally and bilaterally toward the first ribs, ultimately forming a cartilaginous continuity with the first ribs. This bilateral domain extending from the posterior end of the IC mesenchyme to the site of T1 attachment was referred to as the intercostoclavicular mesenchyme (ICC). We adopt the IC and ICC terminology below to indicate the anterior-posterior organization of the presternum. The complex composed of the first appears to join posteriorly with the sternal bars, making up the region of the presternum between the T1 and T2 ribs. Throughout development, the mesenchymal condensations that contribute to the presternum were found to have distinct associations with developing muscles, further supporting their different identities. Distinct muscles from the infrahyoid group were found to be adjacent to the IC or ICC mesenchyme from the earliest stages observed, while the pectoralis was associated with the sternal bars.

**FIGURE 4:**
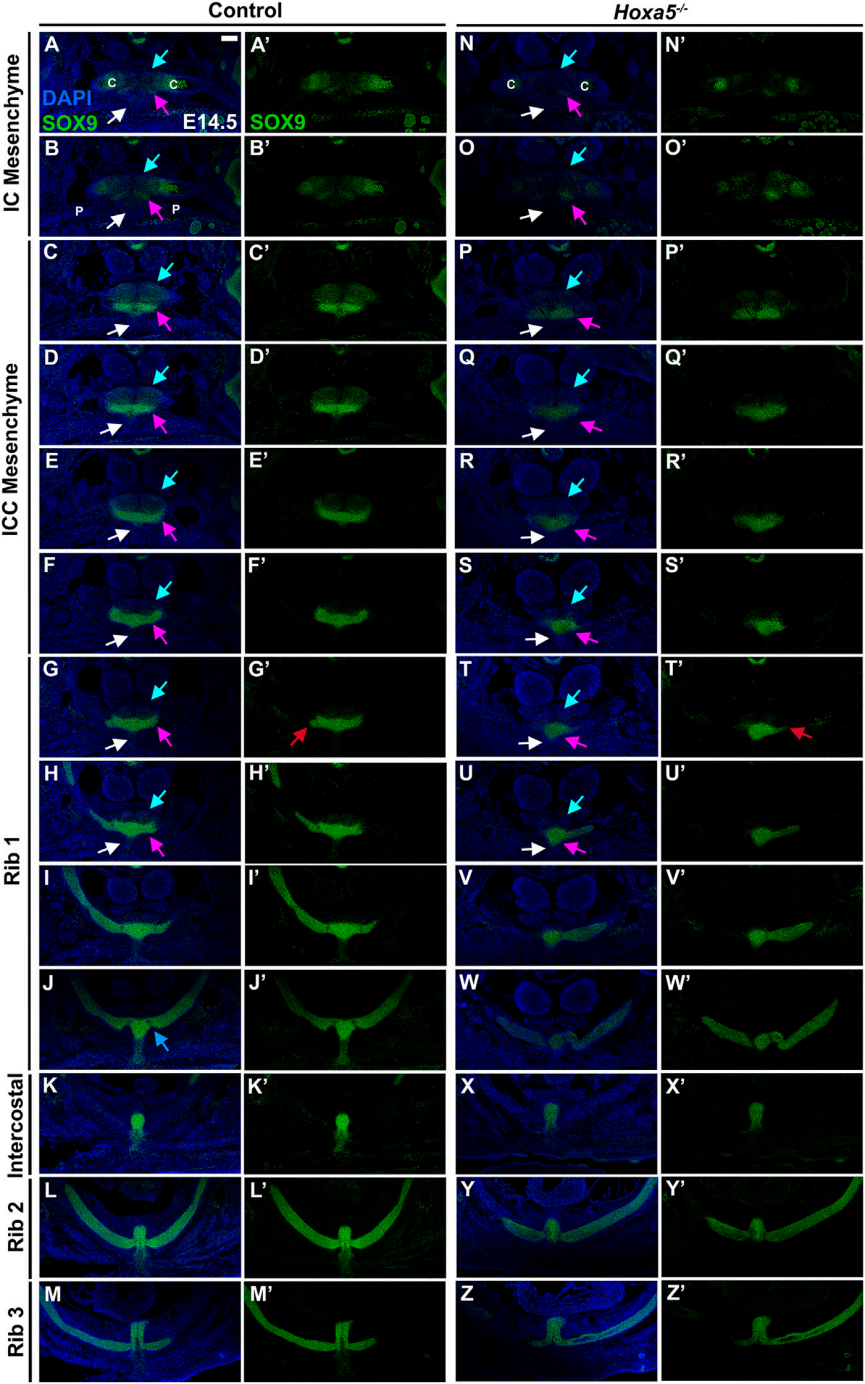
Development of the presternum is disrupted in *Hoxa5* null mutants. **(A**–**M′)** SOX9 expression, shown with **(A–M)** and without **(A′–M′)** DAPI labelling, in serial sections from the anterior presternum through T1–T3 attachments of an E14.5 WT embryo. **(N,N′**–**Z,Z′)** SOX9 expression in serial sections through the presternum and T1–T3 attachment of a Hoxa5^−/−^ null littermate. **(A**–**H)** and **(N**–**U)** Blue arrows mark dorsal IC/ICC; pink arrows indicate ventral ICC; white arrows point to the site of pectoral muscle attachment. Red arrows in **G**′ and **T**′ indicate first contact of T1 with the sternum. Blue arrow in **J** indicates the beginning of the second contact of T1 with the sternum. Scale bar: 200 μM. C, clavicle; IC, interclavicular; ICC, intercostoclavicular; P, pectoral muscle.

To determine whether the mouse presternum develops in a similar manner, we first examined embryos at E14.5, when sternal bar closure is mostly complete. Using Sox9 expression to mark cartilage condensations, we analyzed serial cross-sections, starting at the point of contact between the clavicles and sternum (blue bar in Figure 3K and diagrammed in Figure 3L), and continuing through attachment of the T3 rib. Results are shown in Figure 4. We were able to identify the presternum regions corresponding to the IC and ICC mesenchyme previously described in human embryos (Rodríguez-Vázquez et al., 2013). At the axial level of the IC (Figures 4A,B), Sox9 marks clavicle as well as sternal structures. At this anterior-most point, the presternum appears to contain at least three components: paired dorsal elements that contact the clavicles (blue arrow marks the right side of the pair); separate paired elements that lie ventral to the clavicle (pink arrow marks the right side), and a ventral-midline element where pectoral muscle attachment occurs (white arrow). All three elements continue posterior to the clavicles, in the presternum ICC mesenchyme (Figures 4C–F, arrows as described above). At the axial level of the ICC, the ventral paired elements are larger and bar-shaped, and the level of SOX9 expression within them is increased compared the level of SOX9 observed in the dorsal IC and ICC mesenchyme (compare dorsal and ventral expression in Figures 4A–F). Moving posteriorly through the ICC mesenchyme, these ventral bar-shaped elements change shape, with the lateral ends curving dorsally and the medial portion forming a bulge (Figure 4F) that is more similar in shape to the mesosternum. In contrast, the paired dorsal ICC elements remain rounded and composed of a less dense mesenchyme with lower SOX9 expression compared to the ventral element. In Figure 4G, rib 1 is seen making first contact with the ventral ICC element (red arrow in Figure 4G′). As observed in the adult skeleton (Figures 3I,J), rib 1 makes a double contact. The first is with this ventral element, while the second, more posterior contact occurs in the position where the sternum shifts to a more rounded morphology and no longer contains laterally-extended bars (Figures 4I,J; blue arrow in 4J indicates where the second attachment begins to form. See also Figure 3K). The rounded sternum morphology continues posterior to T1 (Figures 4J–M), including the first intercostal region where the sternum is rounded dorsally and is elongated in the dorso-ventral plane relative to more anterior regions (Figure 4K). This elongated region evidently corresponds to paired, fusing sternal bars at points of rib 2 (Figure 4L) and rib 3 (Figure 4M) attachment. Continuity between ventral ICC and the posterior mesosternum suggests that ventral ICC might represent the anterior ends of the sternal bars.

Transplantation and Prx1-Cre lineage-labeling has shown that the sternal bars are LPM-derived (Bickley and Logan, 2014; Chevallier, 1975; Durland et al., 2008; Fell, 1939) but the origins the presternum components described above are not known. Using the same Prx1-Cre transgene, we found that all of these presternum elements are primarily, if not entirely, of LPM origin (Supplementary Figure S2). We observed Prx1-Cre-RFP in the clavicles (Supplementary Figures S2A”,B”), in the dorsal and ventral elements of the presternum (Supplementary Figures S2C”–J”), and in the mesosternum (Supplementary Figures S2K”,L”; see SOX9 staining in Supplementary Figure S2 for identification of each cartilage condensation). These observations do not exclude contribution to the IC/ICC mesenchyme and clavicles from other tissue sources, and neural crest was previously reported to contribute to the clavicle and manubrium (Matsuoka et al., 2005). However, we were able to rule out any somitic contribution to the presternum by performing a similar analysis with Meox1-Cre (Figures 6,7 and data not shown).

Having identified presternum and T1 skeletal phenotypes in *Hoxa5* mutants, we next examined *Hoxa5* null embryos at E14.5 (Figures 4N–Z, *n* = 4), with references to the condensations described above. While development of the clavicles and IC mesenchyme was normal (Figures 4N,O, blue arrows), the ventral paired elements of the ICC mesenchyme were noticeably smaller than those of littermate controls (compare Figures 4E,F to Figures 4R,S, pink arrows). In contrast, the medial portion of the ventral ICC appears intact. The T1 rib contact is thus perturbed: instead of attaching to a bar of SOX9-positive ventral ICC mesenchyme, which is absent in *Hoxa5* nulls (Figure 4G), rib 1 appears to make contact with the rounded, medial ICC. In this particular *Hoxa5* null specimen, the T1 ribs attach symmetrically and at approximately their normal position on the presternum, comparable to the skeletal examples shown in Figures 3Biii. Moving further posteriorly, the medial element where rib 1 attaches in control embryos becomes more mesosternum-like in its morphology (Figures 4I,J). Here, the *Hoxa5* null sternum remains misshapen (Figures 4V,W). By contrast, the morphology of the sternum at intercostal (Figure 4X), rib 2 (Figure 4Y), and rib 3 (Figure 4Z) levels is similar to those of the control (Figures 4K–M). To determine whether changes in either cell death or cell proliferation were responsible for the reduction of the lateral portions of the ventral ICC in *Hoxa5* mutants, we examined expression of cleaved Caspase 3 and PCNA in E14.5 embryos, but observed no noticeable differences in either (Supplementary Figure S5; *n* = 4).

Together, analysis at E14.5 reveals that *Hoxa5* null embryos have altered sternum morphology from an early stage of development, even in specimens with a relatively normal T1 attachment position. Specifically, the lateral bars of the ventral ICC element are reduced, and their contact with rib 1 is perturbed. In contrast, the IC, dorsal ICC, and all elements posterior to the T1 rib appear normal.

### HOXA5 is Expressed in Ventral ICC of the Presternum at and Anterior to the Site of Rib 1 Attachment

**FIGURE 5:**
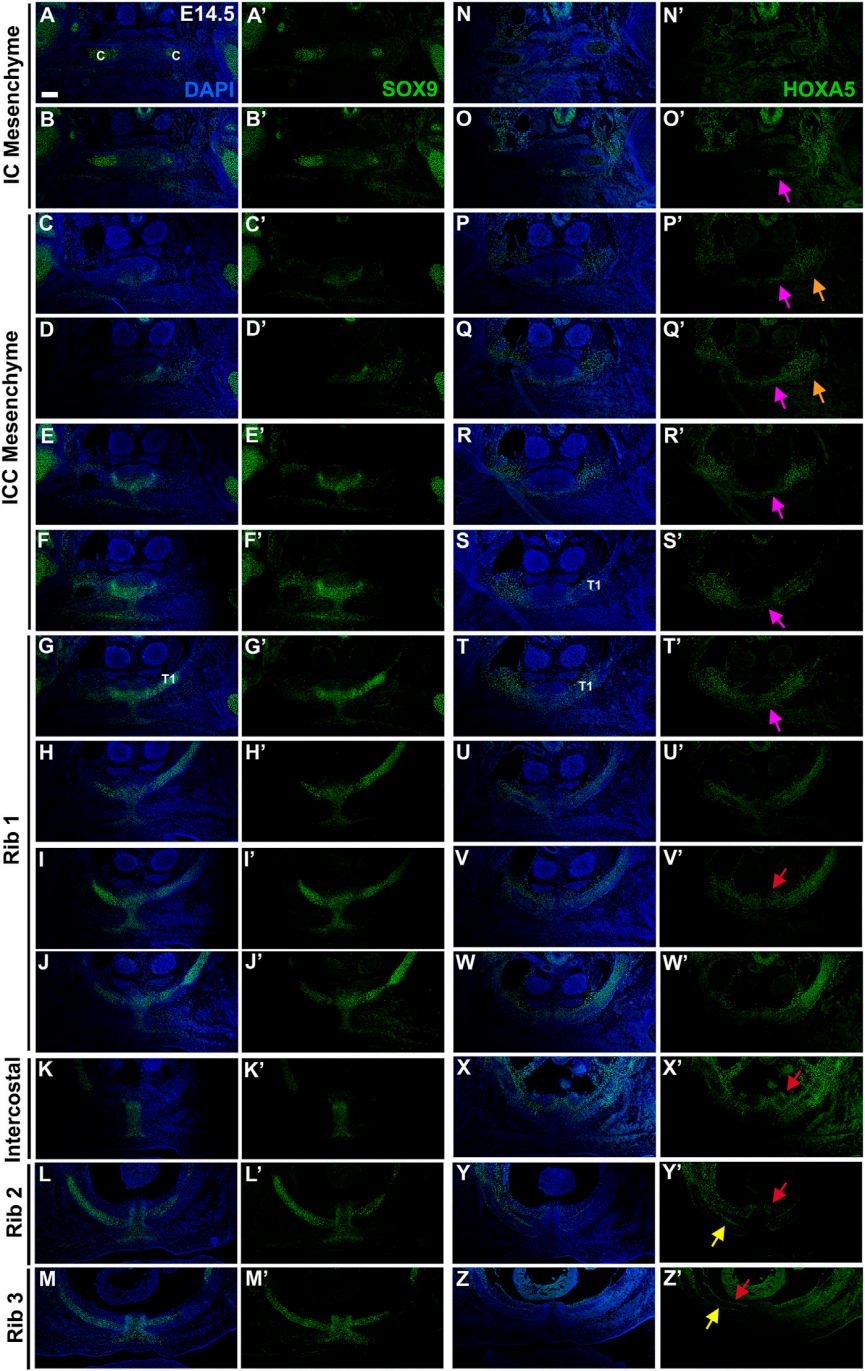
HOXA5 is specifically expressed in ventral elements of the presternum. SOX9 **(A**–**M**′**)** and HOXA5 **(N**–**Z**′**)** expression in alternate sections of a WT E14.5, from the anterior presternum through T1–T3 attachments, shown with **(A–M, N–Z)** and without **(A′–M′, N′–Z′)** DAPI. C, clavicle. Pink arrow in **(O**′–**T**′**)** identifies expression of HOXA5 in the paired ventral elements of the ICC mesenchyme. Orange arrows in **P**′ and **Q**′ mark HOXA5 expression lateral to, but continuous with, the presternum. Red arrow in **V**′ indicates HOXA5 expression within the sternum as it shifts to a more rounded morphology. Red arrows in **X**′ and **Y**′ point to a shift in Hoxa5 expression more dorsally, likely marking the connective tissue of an infrahyoid muscles. HOXA5 can also be seen in the perichondrium of ribs 2 and 3 (yellow arrows in **Y**′ and **Z**′), and in the body wall mesothelium (red arrow in **Z′**). Scale bar: 200 μM. C, clavicle; IC, interclavicular; ICC, intercostoclavicular; T1, thoracic rib 1.

Having determined how *Hoxa5* loss of function affects presternum development, we next inquired whether these phenotypes correspond to a domain of HOXA5 expression in LPM-derived sternal elements. HOXA5 expression was examined in E14.5 embryos and compared to SOX9 in alternate sections from the same embryo (Figure 5) or by double labelling (Supplementary Figure S3). Sternal HOXA5 expression is first detected faintly in the paired ventral condensations at the axial level of the IC mesenchyme (pink arrow, Figure 5O′ and Supplementary Figures S3B,H), and then intensifies within the ICC mesenchyme of the ventral sternum (Figures 5P′–S′; Supplementary Figures S3C,D,I,J). We confirmed that this HOXA5 expression domain was derived from LPM by comparing expression of HOXA5 to Prx1-Cre-RFP (Supplementary Figure S2). Interestingly, while SOX9 marks the entire ventral ICC, HOXA5 labels the lateral edges but is excluded from the midline-most domain (compare Figures 5E′,R′, red arrows in Supplementary Figures S3C′,D′). This pattern of midline exclusion of HOXA5 continues, even as the ventral sternum shifts to a more rounded, mesosternum-like shape at the anterior point of T1 attachment (Figure 5T′). The expression domain of HOXA5 encompasses the same lateral portions of the ventral ICC that appear reduced in *Hoxa5* mutant embryos (Figures 4R,S). At all anterior-posterior levels of the presternum, HOXA5 is excluded from the SOX9-expressing dorsal IC and ICC elements (blue arrows in Figure 4 and Supplementary Figure S3).

In addition to the ventral ICC, HOXA5 is expressed in a domain lateral to but continuous with the developing sternum (Figures 5P′,Q′ and Supplementary Figures S3C,D orange arrows) throughout the ICC region between the clavicle and T1 rib. These HOXA5-expressing cells are also LPM-derived (Supplementary Figure S2F‴, red arrow), and may either reflect cells that are migrating toward incorporation into the presternum (discussed below), or cells marking LPM-derived connective tissue anterior to rib 1. Interestingly, it is this component of the presternum that is elongated in *Hoxa5* mutants, and forms an ectopic ossification following LPM-specific deletion (Figures 3Di,ii,E). Initiation of rib 1 attachment is seen in Figure 5T. Importantly, both rib 1 and the ventral presternum express HOXA5 (Figure 5T′), but while expression looks continuous, the sternal HOXA5 domain is LPM-derived, and the rib 1 domain arises from the somites (compare Supplementary Figures S2H”,H‴).

Expression of HOXA5 in the ventral-lateral ICC extends throughout the ICC and T1 rib contact. Posterior to this, a dorsal LPM expression domain is also observed surrounding infrahyoid muscles at the T1, intercostal and T2 levels (Figures 5V′–Z′, red arrows and Supplementary Figure S2L′, yellow arrow). These muscles were previously described to associate with ICC mesenchyme (Rodríguez-Vázquez et al., 2013). HOXA5 can also be detected in the perichondrium of ribs 2 and 3 (Figures 5Y′,Z′, yellow arrows), and in a thin layer of expression along the body wall mesothelium at the level of rib 3 (Figure 5Z′, red arrow). Importantly, HOXA5 is not expressed in any part of the sternum posterior to the T1 site at E14.5.

**FIGURE 6:**
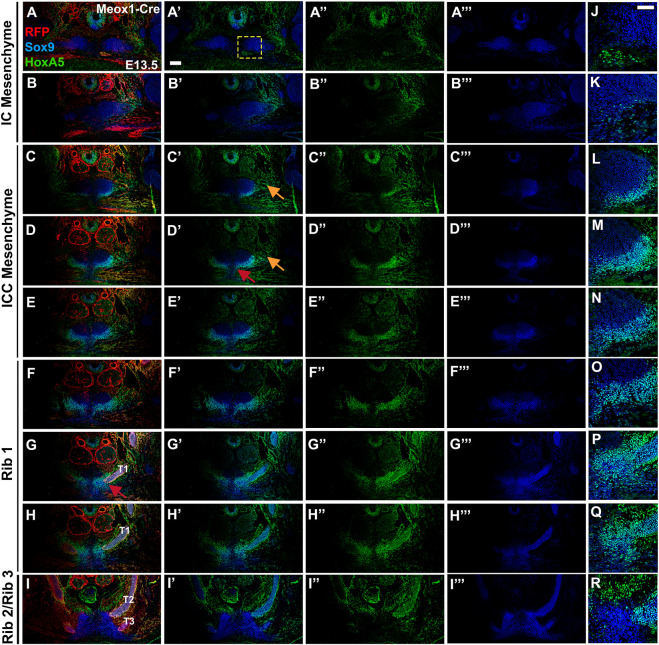
At E13.5, HOXA5 and SOX9 co-expression identifies a component of the presternum that is continuous with the sternal bars, and is the site of rib 1 attachment. **(A**–**I‴)** E13.5 Meox1-Cre-RFP embryo co-labelled for HOXA5 (green **(A′,A**″–**I′**), SOX9 (blue, **(A′,A**‴–**I′,I**‴**)**), and RFP (red **(A–I)**). As observed at E14.5, HOXA5, and SOX9 are co-expressed (cyan cells, **(A,A′–I,I′)** in the ventral ICC element of the presternum—with HOXA5 expression extending more broadly ventral to SOX9 **(C**′–**E**′; **L**–**N)**. **(J–R)** At higher magnification (corresponding to yellow square in **(A**′**)**), co-expression is more clearly visualized. SOX9, but not HOXA5, is detected at lower levels in the dorsal IC and ICC **(A**′–**E**′**)**, while both are co-expressed in ribs 1 and 2 **(F**′–**I**′**)** and spanning the site of rib 1-to-sternum fusion **(F**′–**H**′; **O**–**Q)**. HOXA5 is additionally found in the perichondrium of ribs 1 and 2 **(G**′–**I**′**)**, as well as in the connective tissue of the infrahyoid muscles, dorsal to the sternum **(I**″**)**. Red arrow in **(D**′**)** indicates exclusion of HOXA5 from the most medial region of the ventral ICC mesenchyme. Orange arrows in **(C**′**,D**′**)** point to portion of HOXA5 lateral domain that does not co-express with SOX9. Red arrow in **(G)** indicates site of rib 1 attachment. Scale bars: 200 μM T1, thoracic rib 1; T2, thoracic rib 2; T3, thoracic rib 3.

We next characterized earlier stages of presternum development, including the elements identified above, and tracked both HOXA5 and SOX9 expression in this region. At E13.5 (Figure 6 and Supplementary Figure S4) all of the presternal condensations observed at E14.5 can be distinguished. In the ventral ICC element, SOX9 expression reveals paired bar-like condensations similar to E14.5 except that fusion is apparently not yet complete (red arrows, Figure 6D′ and Supplementary Figure S4E), and thus the medial bulge of the ventral ICC is not present. However, contact between rib 1 and with the ventral ICC bars has already occurred (Figure 6G‴; Supplementary Figure S4F). Interestingly, sternal bar fusion is also incomplete at this stage, thus the transition during rib 1 attachment from the ventral element of the presternum to a more mesosternum-like, sternal bar structure is quite distinct: in Figure 6H‴ and Supplementary Figure S4H, the sternum appears as unfused sternal bars, with a loose collection of SOX9-expressing cells extending between them. And more posteriorly, ribs 2 and 3 attach to unfused sternal bars also connected by a stream of SOX9-positive cells (Figure 6I‴; Supplementary Figures S4I,J). As at E14.5, all presternum elements were composed largely if not entirely from Prx1-Cre labelled, and thus LPM derived cells (Supplementary Figures S4K–T), and none of these presternum elements showed contribution from Meox1-Cre labelled somites (Figures 6A–I).

A comparison of SOX9 and HOXA5 coexpression (Figures 6A′–I′,J–R) or in alternate sections at E13.5 (Supplementary Figures S4K–T) also revealed a pattern similar to that observed a day later at E14.5, with HOXA5 expression largely absent from IC mesenchyme but observed in the ventral ICC mesenchyme of the presternum, where it is localized to a lateral domain that does not express SOX9 (orange arrows, Figures 6C′,D′ and Supplementary Figures S4M‴,N‴) and is LPM-derived (orange arrow, Supplementary Figures S4N′, S6B). To determine whether this lateral domain might include cells that contribute to the connective tissue components of the developing sternum, we co-labeled E14.5 embryo sections for HOXA5 and either Tenascin, a marker of the extracellular matrix associated with tendons and ligaments (Supplementary Figure S6A′) or EBF3, a transcription factor expressed in connective tissue of the developing sternum (Kuriki et al*.,* 2020) (Supplementary Figure S6B′). HOXA5 and Tenascin were not expressed in the HOXA5-expressing cells lateral to the IC/ICC mesenchyme of the presternum (Supplementary Figure S6A, orange arrow, and Supplementary Figure S6A′), suggesting these cells do not correspond to tendon or ligament progenitors. By contrast, HOXA5 and EBF3 are coexpressed in these lateral cells (Supplementary Figure S6B, orange arrow). While this may indicate future contribution of these cells to the sternum based on EBF3 loss-of-function phenotypes (Kuriki et al*.,* 2020), we also observe that EBF3 is expressed broadly in muscle connective tissue (within LPM in Supplementary Figure S6B, and data not shown). HOXA5 has also been previously shown to be expressed in muscle connective tissue (Holzman et al., 2018), thus raising the possibility that this lateral domain acts in establishing the muscle connective tissue of intercostal muscles.

The anterior of the two Rib 1 contacts with the presternum occurs within this region of the HOXA5-expressing ventral-lateral ICC (red arrows, Figure 6G and Supplementary Figure S4P‴). Further, like at E14.5, Rib 1 contact with the presternum continues posteriorly into the region where sternal morphology shifts to unfused sternal bars (Figure 6H‴; Supplementary Figure S4Q‴). At this axial level, HOXA5 expression is also present dorsally and thus surrounds the point of rib 1 contact (Figure 6H”; Supplementary Figure S4R‴), similar to E14.5. However, it does not mark the remainder of the sternal bar. Continuity of SOX9 expression again suggests that the ventral ICC likely represents the anterior-most portion of the sternal bars.

In summary, E13.5 analysis showed the same elements and HOXA5 expression pattern as at E14.5 and additionally showed that the T1 ribs contact the presternum at a transitional point, encompassing both ventral ICC and sternal bars and the junction between them. This region is HOXA5-positive and LPM-derived. Further, at this stage it is clear that pre-sternal elements arise as paired progenitors that flank the midline.

### HOXA5-Expressing Ventral Presternum may Arise From a Lateral Population of Cells

**FIGURE 7:**
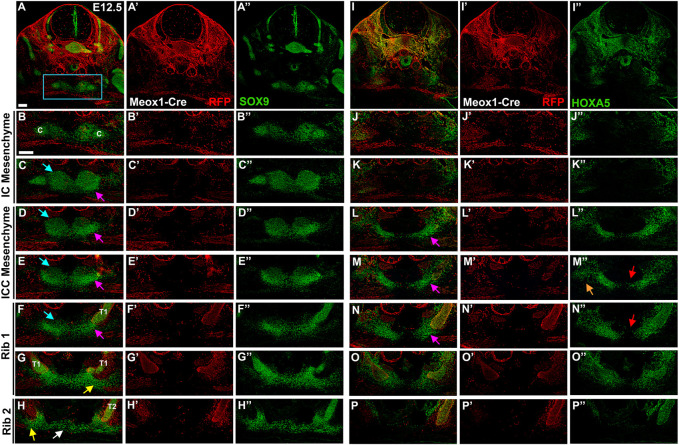
At E12.5, HOXA5 occupies a lateral domain as well as marking the site of rib 1 attachment to the sternum. E12.5 Meox1-Cre-RFP embryo in which alternate sections were labelled for SOX9 **(A**–**H**′′**)** or HOXA5 **(I**–**P**′′**)**. Whole cross-sections are shown in **(A)** and **(I)**, with remaining images from the region indicated by blue rectangle in **(A)**. Images are shown with RFP and either SOX9 **(A**–**H)** or HOXA5 **(I**–**P)**; RFP alone (**A**′–**H**′; **I**′–**P**′), or SOX9 **(A**″–**H**″**)** or HOXA5 **(I**″–**P**″**)** alone. Pink arrows in **(C**–**F)** and **(L**–**N)** indicate ventral ICC. Blue arrows in **(C–F)** point to dorsal IC/ICC. Orange arrow in **(M**″**)** indicates lateral HOXA5 expression domain. Red arrows in **(M**″**,N**″**)** point to exclusion of HOXA5 from the medial-most region of the ventral ICC mesenchyme. Yellow arrow in **(G,H)** marks triangle of SOX9 expression contacting the rib. White arrow in **(H)** marks stream of cells crossing the midline. Scale bar: 100 μM. C, clavicle; T1, thoracic rib 1; T2, thoracic rib 2.

**FIGURE 8:**
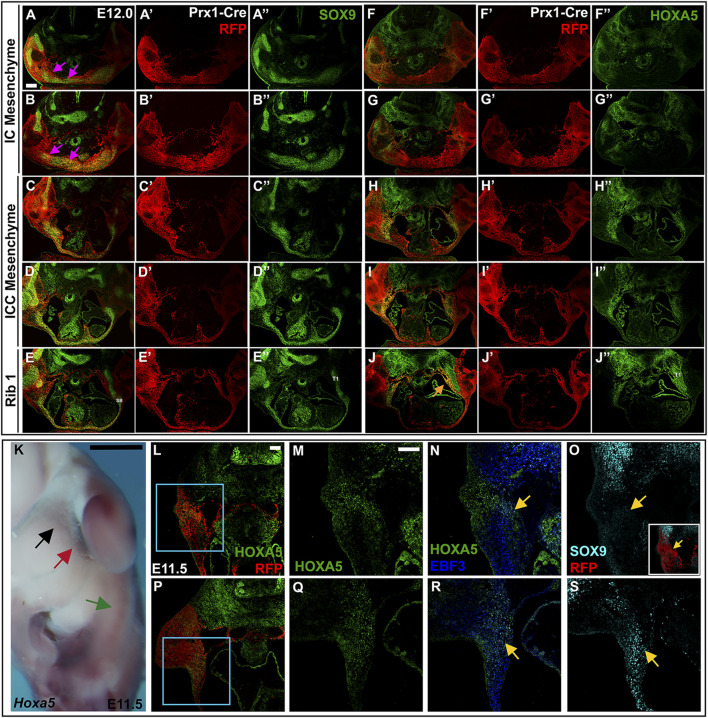
LPM-derived HOXA5 expression can be seen laterally as early as E11.5 and E12. **(A**–**J**″**)** E12.5 Prx1-Cre-RFP embryo in which alternate sections were labelled for SOX9 **(A**–**E**″**)** or HOXA5 **(F**–**J**″**)**. Images are shown with RFP and either SOX9 **(A**–**E)** or HOXA5 **(F**–**J)**; RFP alone **(A**′–**E**′; **F**′–**J**′**)**, or SOX9 **(A**″–**E**″**)** or HOXA5 **(F**″–**J**″**)** alone. Pink arrows in **(A,B)** indicate SOX9 expression associated with developing clavicles and IC elements. **(K)** Whole mount *in situ* hybridization for *Hoxa5* in an E11.5 embryo. Black arrow indicates LPM expression anterior to the forelimb; red arrow points to LPM expression adjacent to the forelimb; green arrow marks additional HOXA5 expression that remains untranslated. **(L,P)** RFP and HOXA5 expression in sections through an E11.5 Prx1-Cre RFP embryo at the level indicated in **(K)** by black arrow **(L)**, or red arrow **(P)**. **(M,N)** expression of Hoxa5 **(M)** or EBF3 and HOXA5 **(N)** in the area indicated by blue square in **(L)**. **(O)** SOX9 expression in an alternate section of same embryo shown in **(L)**. **(Q,R)** expression of HOXA5 **(Q)** or EBF3 and HOXA5 **(R)** in area indicated by blue square in **(P)**. **(S)** SOX9 expression in an alternate section of same embryo shown in **(P)**. Yellow arrow in **(N,R)** indicates region of HOXA5 and EBF3 co-expression. Yellow arrow in **(O,S)** shows corresponding region of SOX9 expression. Inset in **(O)** shows SOX9 expression in the context of Prx1-Cre-RFP. Scale bar: **(A**–**J)**, 200 μM; **(K)**, 25 mm; **(L**–**S)**, 100 μM. SB, sternal bar; T1, thoracic rib 1.

Observation that the presternum appears to be composed of multiple paired elements at E13.5 and E14.5, some of which express HOXA5 and some of which do not, led us to further examine sternum development at earlier stages, including E12.5 (Figure 7), E12 (Figure 8), and E11.5 (Figure 8), with the goal of identifying the axial source of these individual primordia. At E12.5, SOX9 expression in the presternum is very similar to that of E13.5 (Figures 7A”–H”): expression is seen in separate dorsal and ventral presternal elements (Figures 7B”–E”, blue and pink arrows, respectively). Rib 1 contacts the paired ventral ICC elements that have not yet fused or fully condensed into the bar shape observed later (Figure 7F, pink arrow). More posteriorly, the site of rib 1 attachment transitions to the plow-shaped, dorso-ventrally elongated morphology characteristic of the sternal bars (Figure 7G, yellow arrow)*.* Rib 2 similarly contacts the unfused sternal bars, and the bars are connected by a stream of Sox9-expressing cells (Figure 7H, white arrow). Meox1-Cre dependent expression of RFP marks all cells derived from the somites, demonstrating that only the ribs are somite derived, while all components of the sternum are RFP-negative (Figures 7A–H).

HOXA5 immunofluorescence in alternate sections of the same embryo revealed expression in a domain laterally adjacent to but not overlapping the SOX9 positive IC mesenchyme (Figure 7J). Further, this domain of HOXA5 expressing cells is LPM-derived (not shown) and is not labeled with Meox-Cre (Figure 7J′). In contrast, posterior to the clavicle in the ICC mesenchyme, HOXA5 is strongly expressed in the ventral elements of the ICC (Figure 7L”) but remains excluded from the dorsal ICC elements. A comparison with SOX9 reveals that as for later stages, HOXA5 expression is excluded from the most medial cells of the ventral ICC, but overlaps with SOX9 in the lateral part of these condensations (Figures 7M”,N”, pink arrows). In addition, HOXA5 is expressed in a LPM-derived lateral domain that does not stain for SOX9 (orange arrow, Figure 7M”). The SOX9-positive and negative domains of HOXA5 expression here appear continuous, suggesting that the lateral population of HOXA5 positive cells may migrate medially to populate the ventral presternum, and activate chondrogenesis. This broad lateral domain, inside and lateral to the ventral ICC cartilage condensation is observed anterior to rib 1, as well as throughout the region of rib 1 attachment (Figure 7N). As described above, HOXA5 expression in the sternum is restricted to the region around rib 1 attachment, and shifts dorsally when rib 2 appears (Figure 7P). As observed at later stages, HOXA5 expression suggests an important role in the double attachment of rib 1 to the sternum.

Half a day earlier, at 12 days of development, SOX9 is expressed in a broad LPM-derived domain around the midline, in the area that will form the clavicles and the IC mesenchyme, although these structures are not yet morphologically distinct from each other (Figures 8A,B pink arrows). In the IC and ICC regions, the SOX9-positive mesenchyme cannot be resolved as separate dorsal and ventral domains, as it can at E12.5. Instead, a population of HOXA5-positive cells are located lateral to SOX9 domain at the IC and ICC levels (Figures 8F,G). More posteriorly, HOXA5 expression remains lateral, but includes the lateral-most edges of the SOX9 domain, which now extends in a stream of cells across the midline (Figures 8C,D,H,I). At the level of rib1, HOXA5 marks a population of LPM-derived cells lining the medial edge of the incoming rib (Figure 8J, orange arrow). At this stage, the sternal bars appear as a Sox9-positive triangle of cells in contact with the rib (Figure 8E), with a stream of cells reaching across the midline, as previously described (Figure 8E) (Chen, 1952a). These observations suggest that the HOXA5-expressing ventral ICC presternum may arise from this population of LPM-derived cells that develop lateral to the dorsal sternum elements. However, they do not rule out the possibility that HOXA5 expression initiates in the ventral ICC presternum only after those elements form at the midline.

At 11.5 days of development, prior to ventral body wall closure and sternal bar migration, we detected *Hoxa5* transcripts in the LPM, just anterior to and adjacent to the forelimb bud (Figure 8K, black and red arrows, respectively). More posteriorly, additional *Hoxa5* mRNA was detected (green arrow, Figure 8K), but it was previously shown that this corresponds to alternative *Hoxa5* transcripts that are not translated, and no HOXA5 protein is produced posteriorly (Coulombe et al., 2010). In section, LPM-derived HOXA5 expressing cells were observed anterior to the forelimb (Figures 8L,M, black arrow in Figure 8K). Counterstaining was performed for EBF3, a transcription factor previously shown to be expressed in LPM-derived connective tissue precursors as early as E10.5, and required within the LPM for sternum ossification (Kuriki et al., 2020). This revealed co-expression of HOXA5 and EBF3 in a subdomain of HOXA5-positive cells (yellow arrow, Figure 8N). This raises the possibility that HOXA5 marks the progenitors of connective tissue associated with the rib-sternum attachment point in addition to the prospective cartilage itself*.* In an alternate section, a low level of SOX9 was detected in the same region (yellow arrow, Figure 8O). At the level of the forelimb bud (red arrow in Figure 8K), a similar pattern was found. LPM-specific expression of HOXA5 was seen ventral to the forelimb (Figures 8P,Q), a region previously shown to contain sternal bar progenitors (Bickley & Logan, 2014). Co-expression with EBF3 was again observed in a subset of the HOXA5 domain (yellow arrow, Figure 8R), and in an alternate section, SOX9 was expressed in this region as well (yellow arrow, Figure 8S). While we cannot yet determine if either or both of these HOXA5 domains will ultimately contribute to development of the *Hoxa5*-dependent ventral presternum domain that arises later in development, it is clear that LPM-specific expression partially overlaps both SOX9 and EBF3 as early as E11.5.

## Discussion

In this work, we characterized and compared the roles of *Hoxa5* in somites versus LPM, in order to address the broader question of how skeletal development is coordinated between these tissues. Our results led to an analysis of presternum development, which had not been well characterized in mice. The presternum is both morphologically and developmentally distinct from the mesosternum. Anteriorly, it encompasses the sternoclavicular joint--a structure that provides the single point of skeletal articulation between the shoulder girdle and axial column via contact with the clavicles--as well as the attachment site for rib 1. In humans, this clavicle-presternum-T1 unit is further strengthened by the presence of the costoclavicular ligament, which attaches the clavicles to T1. Here, we first present a model for mouse presternum development. In following sections, we discuss evidence that HOXA5 expression marks the embryonic precursor of a previously identified lateral presternum structure that is variably present in therians (Buchholtz et al., 2020) and discuss *Hoxa5* phenotypes with reference the model for presternum development, as well as the combinatorial action of other *Hox* genes. Finally, implications for understanding sternum evolution in the mammalian lineage are discussed.

### The Mouse Presternum Develops From Multiple LPM-Derived Elements

**FIGURE 9:**
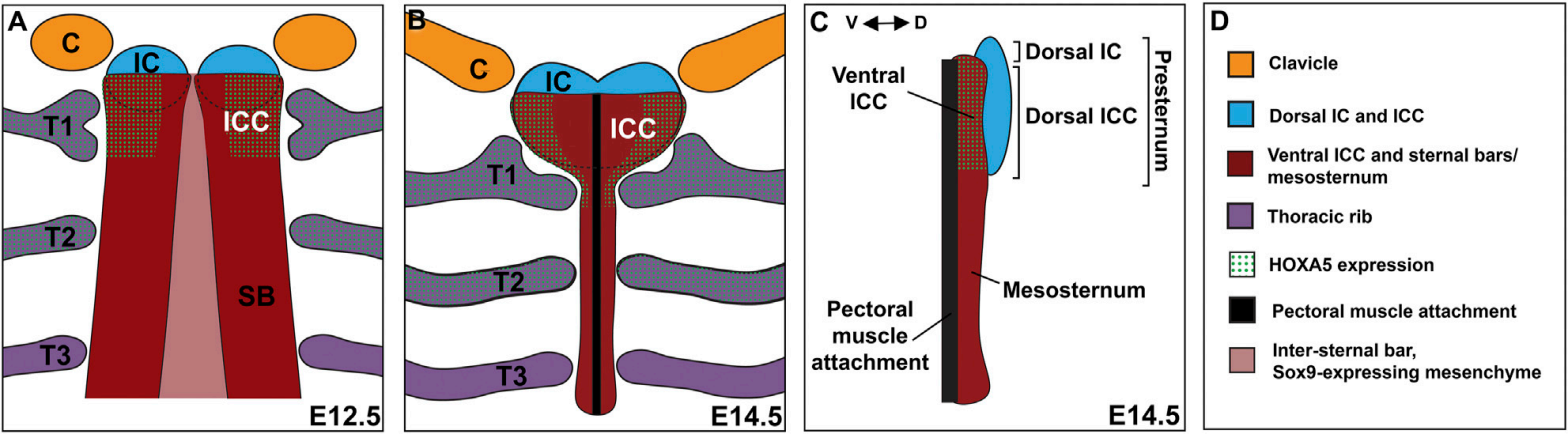
The mammalian presternum is a composite structure with dorsal-ventral organization and molecularly distinct domains. **(A)** Schematic of sternum development in an E12.5 embryo. The clavicles (orange) and dorsal IC/ICC mesenchyme form as paired condensations that express low levels of SOX9. The ventral ICC of the presternum appears as a region of high SOX9 expression that is continuous with the posterior sternal bars (SB). HOXA5 is expressed in both the ventral ICC and rib 1, marking the site of rib 1 attachment to the sternum. Rib 2 also expresses high levels of HOXA5. SOX9 expression can be seen in a stream of cells that cross the midline between the sternal bars. **(B)** Schematic of sternum development in an E14.5 embryo. By E14.5 sternal bar fusion is mostly complete and the presternum can be seen to have a morphology distinct from the rest of the sternum. The clavicles articulate with the dorsal IC, while the first rib contacts the HOXA5-expressing ventral ICC in two places. The pectoral muscles attach along the ventral midline of the sternum. **(C)** Side view of an E14.5 sternum showing dorsal ventral regionalization. The dorsal IC and ICC (blue) form a distinct element, while the ventral ICC appears continuous with the sternal bars. HOXA5 specifically marks the ventral ICC. **(D)** Key for coloring in **(A**–**C)**.

Analysis across a time-series allowed a detailed description of presternum development in mouse. We propose that there are at least five elements contributing to the presternum, each of which arises as paired primordia and is derived primarily from LPM with no contribution from the somites. Further, we propose that there are at least two progenitor domains for these five elements (and more are possible). This model is summarized in Figure 9. The existence of an additional, lateral and LPM-derived progenitor domain contributing to the presternum at the point of T1 attachment is also supported by our results (see below).

By E14.5, five SOX9-positive condensations of the presternum were observed (Figures 9B,C): 1) the IC mesenchyme between the clavicles continuous with 2) dorsal ICC mesenchyme, which is a heart-shaped, loose mesenchyme anterior to and flanking the T1 rib attachment; 3) ventral ICC mesenchyme, which is a bar shaped dense mesenchyme anterior to and flanking the T1 rib; 4) ventral midline mesenchyme likely forming the connective tissue of the pectoral muscle attachment. and 5) the sternal bars, which have fused at the midline by this stage and are clearly visible from the posterior edge of the T1 attachment and extending posteriorly through the rest of the sternum. Because the IC and dorsal ICC appear morphologically continuous they are represented as a single dorsal component (colored blue in Figure 9). Similarly, the ventral ICC is morphologically continuous with the sternal bars and they are represented as a second component (colored red in Figure 9).

Several points from past and the present work support these two regions as having distinct developmental origins. Indeed, other descriptions of presternum development in mammals agreed that the presternum incorporates anterior sternal bars (Gladstone & Wakeley, 1932; Rodríguez-Vázquez et al., 2013) and references therein). Further, the anterior presternum reportedly consists of dermal bone while the more posterior part of the presternum is endochondral, like the mesosternum (Gladstone & Wakeley, 1932). The former region has been proposed to be homologous to the interclavicle, which is a midline component of the presternum in most tetrapods, but absent in therian mammals (for example, (Gladstone & Wakeley, 1932; Rodríguez-Vázquez et al., 2013; Buchholtz et al., 2020), and references therein).

Our examination at stages prior to E14.5 also suggest at least two progenitor domains for the presternum and extends previous characterizations of the ICC and T1 attachment point. At E11.5, progenitors of sternal bars are known to be clustered in a triangle of cells ventral to the forelimb bud, many of which, as previously reported, express EBF3 and/or SOX9 (Bickley & Logan, 2014; Kuriki et al., 2020). In addition, beginning as early as E11.5 and until fusion of the sternal bars, we observed a continuous line of SOX9-postitive cells reaching across the midline from one sternal bar to the other. These may be the “stream of flattened cells” previously described as migrating with the outgrowing intercostal muscles (Chen, 1952a). No other sternal progenitors are known at that stage. However, at E12, we first observed a paired, SOX9 positive condensation between and largely continuous with the clavicle condensations, and extending posteriorly into the ICC region, similar to what was recently reported in human embryos (Rodríguez-Vázquez et al., 2013). At this stage, the sternal bars are in a separate and more dorso-lateral location, have already fused with the outgrowing rib anlage, and migrated ventrally from their point of origin in the axillary region. One half day later, at E12.5, all five elements described above are distinct (Figure 9A). A more closely spaced time series between E12-E12.5, coupled with fate mapping, could further resolve the origin of the five regions above.

Observations across this time series indicate that the ICC region at and just anterior to the T1 attachment represents a unique AP domain in which separate dorsal and ventral ICC elements overlap with one another. Further, the lateral-most portion of the ventral ICC is molecularly distinct based on its differential expression with HOXA5 (discussed below).

Additionally, our time series shows that all of these five presternal regions originate from paired progenitor domains, and thus likely migrate toward and/or fuse across the midline. Finally, lineage-labeling with Prx1-Cre shows that all five presternal elements are primarily if not entirely LPM-derived, consistent with previous observations for the sternal bars (Fell, 1939; Chevallier, 1975; Durland et al., 2008; Bickley & Logan, 2014) and the sternum at the point of T1 attachment (Durland et al., 2008). Further, Meox1-Cre labeling shows that none of these elements contain contribution from the somites, which definitively marks the position of the lateral somitic frontier at the points of rib attachment. It was previously reported that post-otic neural crest contributes to the clavicle and anterior presternum (Matsuoka et al., 2005), which is almost certainly restricted to the IC mesenchyme.

### HOXA5 Expression Identifies a Molecularly Distinct Region of the Presternum and T1 Rib Attachment

HOXA5 is expressed in the developing T1-T3 ribs (Figure 5; Holzman et al., 2018). Here we show it is also expressed in the presternum, but specifically at both points of T1 rib contact as well as anterior to rib 1 (green hatching, Figure 9). This domain of expression corresponds to the ventral-lateral ICC. HOXA5 is also expressed in presternum at the second, more posterior point of T1 contact, where there is sternal bar-like morphology. The posterior border of HOXA5 expression in the sternum marks the posterior edge of the T1 attachment. Finally, in addition to expression in these lateral presternum cartilage condensations (as marked by SOX9 expression), at E12.5–E13.5 we also observed HOXA5 in laterally adjacent, SOX9-negative cells. It is possible that these lateral cells contain progenitors that will contribute to the presternum condensation, or alternatively they may become connective or other tissues of the surrounding sternoclavicular joint. In either case, the position of HOXA5-expressing cells in LPM specifically surrounding the point of rib 1 contact suggests that HOXA5 marks the embryonic anlage of a recently-defined lateral presternum element. This was identified as a separate lateral presternum ossification present at low penetrance in human medical scans, and also variably present in some groups of therians (Buchholtz et al., 2020). These results suggest that the embryonic primordium of this region is present in mice and thus may be common to all therians. HOXA5 expression marks this lateral embryonic element and HOXA5 expression was absent from other regions of the developing presternum (Figure 9).

It is notable that the presternum HOXA5 expression domains spatially coincide so precisely with the HOXA5-expressing T1 ribs. Indeed, expression in the LPM is notably specific to regions immediately adjacent to somite-derived and HOXA5-positive tissue from the earliest (E11.5, Figure 1) to the latest (E14.5, Figure 5) stages we examined. As deletion of *HoxA5* in either LPM or somitic mesoderm affects attachment of the T1 rib, this bridge of HOXA5 expression across the lateral somitic frontier may allow for proper communication between these two distinct but cooperating mesoderm sources. In this context, it would be interesting to know whether expression of *Hoxa5* is co-regulated in somites and LPM. In fact, the cis-regulatory elements that control both forelimb bud and somitic transcription of the single *Hoxa5* coding transcript have been localized to a 2.1 kb fragment termed the mesodermal enhancer (MES) (Larochelle et al., 1999). However, reporter assays suggest that CREs necessary for somitic vs. limb bud expression can be at least partially separated but reside within the same ∼900 bp fragment (Tabariès et al., 2007). Additionally, CDX1 binding sites within the same region define the sharp posterior boundary of somitic expression. It would be interesting to further characterize this CRE from mouse and other mammals, to better understand how this intricate pattern of expression is achieved across the lateral somitic frontier.

### Tissue-Specific Roles for *Hoxa5* in Presternum and Rib Development

Results from conditional deletion of *Hoxa5* in somites vs. LPM show that *Hoxa5* plays largely independent roles in these two tissues: the conditional deletion phenotypes for the most part additively explain the *Hoxa5* null skeletal phenotype.

Somite-specific *Hoxa5* deletion reproduced all of the vertebral (primaxial) skeletal phenotypes associated with the null allele, including homeotic transformations of C7-T1. This included both vertebral changes and a gain of ectopic C7 ribs, which could be unilateral or bilateral, and could be free, fused to the T1 rib, or fused to the sternum. While the proximal ribs are primaxial, the distal T1 rib is abaxial (Durland et al., 2008). However, LPM-specific *Hoxa5* activity was dispensable for T1 (and C7) rib phenotypes including symmetrical guidance and fusion to the presternum.

Somite-specific *Hoxa5* activity is also necessary for proper positioning of the T1 fusion to the presternum, and Meox1-Cre conditional deletion reproduced the asymmetric T1 fusion and T1 bifurcation phenotypes associated with the null allele. As mentioned above, these phenotypes could be explained in at least two ways, which are not mutually exclusive. First, somitic *Hoxa5* activity may be required for rib progenitors (and those of associated intercostal muscle and connective tissue) to migrate segmentally across the lateral somitic frontier. This segmental migration is known to rely on signaling among somite derivatives and to involve guidance molecules such as Ephs/Ephrins (Compagni et al., 2003). Second, it is also possible that symmetrical T1 fusion is achieved because the presternum attachment point is molecularly unique. HOXA5 expression in somitic tissues may be necessary for recognition and attachment to this unique region, and attachment becomes more random in its absence resulting in altered an asymmetric rib fusions. HOXA5 itself could contribute to molecularly defining this point of attachment, although its sternal expression is clearly dispensable for symmetry of T1 fusion. However, it is required for morphogenesis of the presternum; in null embryos, we observed that the lateral, HOXA5-expressing portions of the presternum are misshapen and thus the morphology of this attachment is altered even in embryos with symmetrical rib fusion. Unfortunately, the perinatal lethality of the *Hoxa5* mutation (due to respiratory defects) prevents analysis at stages when this region is further developed.

LPM-specific activity of *Hoxa5* is shown here to include negative regulation of presternum extension anterior to T1; in both null embryos and in those with LPM-specific *Hoxa5* deletion this region was rostro-caudally elongated. The phenotype was more extreme in the latter case. We suspect that it is the ventral ICC that is perturbed, as these are the only LPM-derived cells in the affected region that express HOXA5. HOXA5 could negatively regulate recruitment of mesenchyme to the lateral ICC condensation. Indeed, it is expressed both within the SOX9 condensation and in mesenchyme lateral to it. Alternatively or in addition, HOXA5 could regulate the progress of chondrogenic differentiation which would also alter the morphology of the resulting structure. Interestingly, both roles were previously described for *Hoxa5* in the acromion (Aubin et al., 2002), which is the portion of the scapula that articulates with the lateral end of the clavicle.

This presternum elongation was the only phenotype that may be non-additive. The elongation of the presternum was far more pronounced in LPM-deleted embryos than in *Hoxa5* null embryos; further, in the former, several embryos were observed with a complete additional ossification between T1 and the Y-shaped cartilage. This could not be considered a homeotic transformation by the criterion available at E18.5: in these embryos, the sternum does not segment at the point of T1 attachment, in contrast to mesosternal joints where segmentation is apparent. Rather, this phenotype could arise simply by failure to limit recruitment or proliferation of cells into the ventro-lateral ICC, or to regulate differentiation, as described above. It is possible that the more severe phenotype in conditional compared to complete knockouts results from a requirement for communication between somites and LPM, and that a mismatch in their Hox code exacerbates the effect. However, we cannot rule out contribution of mixed genetic background introduced from the Prx-1-Cre line altering the expressivity of the phenotype. Countering this, the *Hoxa5* skeletal phenotype has been characterized in different backgrounds and in no case were ectopic presternum ossifications ever observed (Aubin et al., 2002). Finally, we cannot totally reconcile two different phenotypes but note they are not mutually exclusive: in whole-mount skeletons there is evident AP elongation of the presternum corresponding to the ventral ICC. In sections, we also observe a truncation in the medio-lateral direction; this is more spatially restricted to the T1 attachment point.

In contrast to the presternum, there was no effect of *Hoxa5* deletion (either conditional or complete) on the length of the mesosternum, consistent with its lack of expression posterior to T3.

Finally, while HOXA5 expression is largely excluded from the IC and dorsal ICC, we noted that the Y-shaped cartilage that develops from this region is often smaller in *Hoxa5* null or LPM-specific mutants. Further, in null embryos both the Y-shaped cartilage and the point of T1 attachment often ossify prematurely. This latter phenotype was not produced by LPM-specific deletion, and only once following somite-specific deletion. This could indicate that either domain of *Hoxa5* is sufficient to regulate ossification of the presternum, or alternatively, that *Hoxa5* acts in a different tissue (such as neural crest) to mediate this phenotype.

### A Presternum Hox Code

It is well established that Hox genes act combinatorially to pattern somites and neural tube derivatives, with nested, colinear expression important for their function (reviewed in (Krumlauf, 2018)). With the exception of the limbs, colinear Hox expression is not well-reproduced in the body wall LPM. However, genetic evidence indicates a combinatorial Hox code is also operating in the presternum.

For example, while *Hoxa5* mutants show an elongated presternum anterior to the T1 rib, in contrast, the entire presternum anterior to the T2 attachment is absent in *Hoxa5; Hoxb5; Hoxc5* triple mutants (McIntyre et al., 2007). This phenotype is consistent with a loss of both the dorsal and ventral ICC elements described here. It is also consistent with other *Hoxa5* phenotypes including homeotic vertebral transformations that show opposite effects in *Hoxa5* single mutants compared to *Hoxb5* or *Hoxa5; Hoxb5; Hoxc5* triple mutants (Jeannotte et al., 1993; McIntyre et al., 2007). Together, this highlights the often antagonistic activity of *Hoxa5* and *Hoxb5*.

The *Hox4* and *Hox6* paralog groups also pattern in the presternum. Following triple knockout of all *Hox6* paralogs, the distance between T1-T2 rib attachments is greatly reduced and fails to ossify, but the Y-shaped cartilage anterior to T1 forms(McIntyre et al., 2007). This could be interpreted as disruption specifically of the ventral ICC posterior to T1, a region that is HOXA5-negative and has a sternal-bar like morphology. In contrast, *Hoxb4* mutants show disruption of the anterior presternum: the Y-shaped cartilage is absent, but 2 strips of unfused cartilage articulate with the T1-T2 ribs (Ramirez-Solis et al., 1993). This could possibly result from disruption of the IC/dorsal ICC, but retention of the ventral ICC (which fails to fuse). All of these *Hox* phenotypes are consistent with this model of presternum development, and combinatorial effects on its different components.

Symmetrical rib attachment to the sternum is a genetically separable phenotype from the growth of the various presternum components. This is shown for *Hoxa5* above, where *Hoxa5* in somites is necessary symmetrical T1 fusion. Although HOXA5 is also expressed in rib 2, *Hoxa5* single knockouts do not have T2 phenotypes. In contrast, in *Hoxb5* single mutants and *Hoxb5; Hoxb6* double mutants, the second rib often bifurcates and one arm fuses at the T1 rib position (in the same animals the T1 rib is either missing or shortened (Rancourt et al., 1995). *Hox9* compound mutants also have aberrant fusion of T2 at the T1 position (McIntyre et al., 2007). Together, this implicates multiple *Hox* genes in positional information located either on the sternum and/or in the migrating distal ribs or associated tissues.

### Implications for Sternum Evolution

The sternum arose in early tetrapod evolution as an adaptation to life on land, and sternum morphology varies with mode of locomotion and respiration, making it a good system in which to investigate the developmental mechanisms underlying evolutionary change. In addition, the sternum is of interest because it is the site of interaction between mesoderm derivatives with different embryonic origins. In this way, the site of contact between the two, the lateral somitic frontier, requires the evolution of signals that function across it.

The mammalian sternum has been reduced in size and skeletal structure number over the course of evolution. On the basis of fossil evidence, it has been proposed that this reduction is either due to the loss of the primitive interclavicle, the ancestral structure that constitutes the entire sternum in basal synapsids, in the transition to basal therians, or to the incorporation of the interclavicle into a single presternum structure that articulates with clavicles anteriorly, rib 1 laterally, and rib 2 posteriorly. Loss or incorporation of the interclavicle has thus been thought of as key step in sternum evolution. Using fossil and anatomical imaging, evidence has been found suggesting that the mammalian presternum is in fact a composite structure containing the ancestral interclavicle, as well as the anterior ends of the sternal bars. In addition, Buchholtz et al (2020) provide evidence for the existence of lateral elements that are the site of rib 1 attachment. Our observations of presternum development in mice support this hypothesis that the mammalian presternum is a composite structure. We show that the presternum has dorsal-ventral organization, and that it contains LPM-derived IC and ICC elements (Figure 8). Our analysis of SOX9 expression indicates that the dorsal IC and ICC may represent the more primitive interclavicle element. The ventral ICC likely arises from the anterior ends of the sternal bars, and represents what is referred to as the manubrium in the fossil record (e.g., Luo et al., 2007), in agreement with previous studies (Rodriguez Vázquez et al., 2013; Buchholtz et al., 2020). Moreover, while morphologically continuous with the sternal bars, analysis of *Hoxa5* function and expression reveals that the ventral ICC is molecularly distinct, and may indeed contain HOXA5-expressing lateral elements that provide the site of rib 1 attachment. Finally, our finding that HOXA5 expression spans the lateral somitic frontier, marking both somite-derived ribs and LPM-derived sternum, suggests that *Hoxa5* may play an important role in allowing for communication between both mesoderm types at the lateral somitic frontier, thereby permitting establishment of the unique rib 1-sternum attachment site.

## Methods

### Mouse Strains and Genotyping

The following mouse strains were used: *Hoxa5-Cre: Tg(Hoxa5-cre)447BLjea* (Bérubé-Simard & Jeannotte, 2014); *Hoxa5* ^*flox*^: *Hoxa5* ^*tm1.1Ljea*^ (Tabariès et al., 2007); *Prx1-Cre*: *B6.Cg-Tg(Prrx1-cre)1Cjt/J* (Logan et al., 2002); *Rosa6* ^*tdtomato*^; *B6.Cg-Gt(ROSA)26Sortm9(CAG-tdTomato)Hze/J* (Madisen et al., 2010); *Hoxa5* ^-^: *Hoxa5tm1Rob* and Meox1Cre: *Meox1tm1(cre)Jpa* (Jukkola et al., 2005). Conditional knockout or RFP-labeling was carried out by crossing a male harboring a Cre allele to a female with a loxP allele. Timed-pregnant females were sacrificed to collect embryos at the stages indicated. Genotyping of tail snip DNA was performed with primers for Cre, RFP, *Hoxa5* ^+^ or *Hoxa5* ^-^ alleles. All procedures were performed in accordance with the NIH Guide for Care and Use of Laboratory Animals and approved by the Columbia University IACUC. Genotyping primers (5′ to 3′) were as follows. Cre: forward GCG​GTC​TGG​CAG​TAA​AAA​CTA​TC, reverse GTG​AAA​CAG​CAT​TGC​TGT​CAC​TT; tdTOMATO/RFP: forward CTG​TTC​CTG​TAC​GGC​ATG​G, reverse GGC​ATT​AAA​GCA​GCG​TAT​CC; *Hoxa5* ^*+*^ allele: forward ACT​GGG​AGG​GCA​GTG​CCC​CCA​CTT​AGG​ACA, reverse CTG​CCG​CGG​CCA​TAC​TCA​TGC​TTT​TCA​GCT; *Hoxa5* ^*-*^ allele forward ACT​GGG​AGG​GCA​GTG​CCC​CCA​CTT​AGG​ACA, reverse GGC​TAC​CTG​CCC​ATT​CGA​CCA​CCA​AGC​GAA.

### Skeletal Staining

Alcian Blue and Alizarin Red staining was performed on E18.5 embryonic skeletons as previously described (Lufkin et al., 1992).

### Immunofluorescence

Immunostaining was performed as described (McGlinn et al., 2019). Briefly, embryos were fixed 2 h to overnight in 4% paraformaldehyde at 4°C and embedded in OCT. 8–10 μm sections were cut, tissue was permeabilized and blocked in 5% Normal Donkey Serum/0.3% Triton-X/PBS and incubated in primary antibodies overnight at 4°C, washed in PBS, and incubated for 3 h RT with Alexa-488 or 594-conjugated secondary antibodies (Jackson Immunoresearch, diluted 1:400). Slides were washed in PBS counterstained with DAPI, and mounted in Prolong Diamond. Primary antibodies: HoxA5 (Phillipidou et al., 2012; 1:5000); Sox 9 (Millipore-Sigma AB5535, 1:500 or RnD Systems AF3075-SP 1: 500); RFP (Chromotek 5F8, 1:1000); Ebf3 (RnD Systems AF5166, 1:1000); Tenascin (Sigma-Aldrich T3413, 1: 100); PCNA (Santa Cruz sc-56 1:200), Cleaved Caspase 3 (CST 9661 1:200).

### *In situ* Hybridization

Whole-mount *in situ* hybridization for *Sox9* was performed as previously described (Brent et al., 2005).

## Data Availability

The original contributions presented in the study are included in the article/Supplementary Material, further inquiries can be directed to the corresponding author/s.

## Supplementary Material

Supplementary Figure 1 Rib and sternum defects following conditional deletion of *Hoxa5.* **(A)** control sternum showing proper T1 and T2 fusion. **(B**–**D)** Examples of rib and sternum phenotypes, described in the text. In all panels, black arrowheads indicate T1 ribs, black arrows indicate T2 ribs, and grey arrowheads indicate (ectopic) C7 ribs. Grey arrow indicates (ectopic) ossified sternebra anterior to T1. White arrowhead in Bii indicates ossification of the Y-shaped cartilage, White arrow in Cv indicates fused sternebrae, also called crankshaft sternum. Most of the whole-mount views shown here correspond to specimens shown in Figure 3 after sternum dissection. Correspondence of images is as follows: Supplementary Figures S1A to Figure 3A, control embryo; Supplementary Figures S1Bi to Figure 3Bii *Hoxa5* null embryo; Supplementary Figures S1Ci-iii to Figure 3Ci-iii; three somite-conditionally deleted embryos; Supplementary Figures S1D to Figure 3Di, LPM conditionally deleted embryo. Specimens shown in Supplementary Figures S1Bii,Civ,v are not shown in Figure 3.

Supplementary Figure 2 Medial clavicles and sternum are derived from LPM. Analysis of E14.5 Prx1-Cre-RFP embryo in which alternate sections were labelled for SOX9 or HOXA5. Images are shown with RFP, SOX9 or HOXA5, and DAPI **(A**–**L)**, without DAPI **(A**′–**L**′**)**, RFP alone **(A**”–**L**”**)**, and with SOX9 or HOXA5 alone **(A**‴–**L**‴**)**. Red arrow in **(F**‴**)** marks lateral HOXA5 expression. Yellow arrow in **(L**′**)** points to LPM-derived connective tissue in an infrahyoid group muscle. Scale bar: 200 µm. C, clavicle; IC, interclavicular; ICC, intercostoclavicular.

Supplementary Figure 3 At E14.5, HOXA5 and SOX9 are co-expressed in the cells of the ventral ICC mesenchyme. **(A**–**F)** E14.5 embryo co-labelled for HOXA5 (green, **A**′–**F**′) and SOX9 (red, **A**”–**F**”). **(G**–**L)**. Higher magnification of the region corresponding to the yellow square in **(A)**, reveals co-expression (yellow cells). Co-expression is seen in the ventral ICC **(I,J)**, with the HOXA5 domain extending more broadly ventral to SOX9. HOXA5 and SOX9 additionally co-express in ribs 1 and 2 **(K,L)** and spanning the site of rib 1-to-sternum fusion **(K)**, while HOXA5 alone is detected in the perichondrium of ribs 1 and 2 **(E,F)**, and in the connective tissue of the infrahyoid muscles, dorsal to the sternum **(E,F)**. Pink arrows in **(B,H)** mark first HOXA5 expression in the ventral mesenchyme. Red arrows in **(C**′,**D**′**)** indicate exclusion of HOXA5 from the most medial region of the ventral ICC mesenchyme. Orange arrows in **(C,D)** point to HOXA5 lateral domain that does not co-express with SOX9. Scale bars: 200 μM.

Supplementary Figure 4 Hoxa5 marks the ventral presternum at E13.5. **(A**–**J′)** SOX9 expression in serial sections of a WT embryo after E13.5 days of development, from the presternum to attachment of rib 3, shown with **(A–J)** or without **(A′–J′)** DAPI staining. All of the presternal condensations observed at E14.5 can be distinguished: the paired dorsal elements are present in IC mesenchyme at the site of clavicle attachment **(A,A′)** and extend posteriorly through the ICC region. Just posterior to the clavicles, the paired ventral elements that express HOXA5 at E14.5 are also visible (pink arrow, **B**) and extends through the ICC region **(C**–**E)**. The ventral-most SOX9-positive mesenchyme, which articulates with pectoral muscles, is also apparent (white arrow, **C**′). Red arrow in **(E)** points to the space between the paired ventral elements that have not yet fused at the midline. To confirm that each presternum element was derived from the LPM, we again compared SOX9 expression to that of RFP under control of Prx1-Cre **(K**–**T)‴**. E13.5 Prx1-Cre-RFP embryo in which alternate sections were labelled for SOX9 or HOXA5. Images shown with RFP, SOX9 or HOXA5, and DAPI **(K**–**T)**, without DAPI **(K**′–**T**′**)**, RFP alone **(K**”–**T**”**)**, and with SOX9 or HOXA5 alone **(K**‴–**T**‴**)**. While the ribs were clearly RFP-negative as expected (R), all SOX9-expressing sternal elements were RFP-positive **(K**–**T)**. As at E14.5, none of these presternum elements showed contribution form somites (not shown). A comparison of SOX9 and HOXA5 in alternate sections at E13.5 revealed a pattern similar to that observed a day later at E14.5. HOXA5 expression is largely absent from the presternum in the IC mesenchyme **(L**‴**)**, but it is observed in the ventral ICC mesenchyme in a lateral domain (orange arrows, **N**′, **N**‴) that also expresses SOX9 (orange arrow, **M**‴) and is LPM-derived (orange arrow, **N**′). Additionally, HOXA5 is expressed in the ventral presternum of the ICC region (pink arrows, **N**‴, **P**‴) anterior to T1. Note: the plane of section in this example is uneven, hence the right side of the embryo is slightly more posterior than the left side. Anteriorly, Rib1 makes contact with the HOXA5-expressing ventral-lateral presternum (red arrow, **P**‴). Like at E14.5, Rib 1 contact with the presternum also continues posteriorly into the region where sternal morphology shifts to unfused sternal bars (**Q**‴). At this axial level, HOXA5 expression is also present dorsally and thus surrounds the point of rib 1 contact (**R**‴). However, it does not mark the remainder of the sternal bar (green arrow, **R**‴) On the embryo’s left side, which is slightly anterior to the right, HOXA5 is expressed in its lateral, LPM-derived domain (orange arrow, **R**′) like at E14.5. **T**‴ shows the sites of rib 1 and rib 2 attachment: sternal HOXA5 marks the point of rib 1 contact on the embryo’s left side (red arrow, **T**‴), but shifts to its more dorsal domain at the point of rib 2 contact (green arrow, **T**‴). Pink arrows in **B**, **N**‴, and **P**‴ mark the ventral ICC that expresses Hoxa5. Orange arrows in **M**‴ and **N**‴ indicate lateral SOX9 or HOXA5 expression. Red arrows in **T**‴ and **P**‴ point to LPM-HOXA5 expression at the point of rib 1 fusion. Green arrow in **T**‴ marks the point of rib 2 attachment, where HOXA5 has now shifted dorsally. Scale bar: 200 μM. C, clavicle; IC, interclavicular; ICC, intercostoclavicular; P, pectoral muscle; SB, sternal bar; T1, thoracic rib 1; T2, thoracic rib 2; T3, thoracic rib 3.

Supplementary Figure 5 Reduction in size of lateral ventral ICC mesenchyme in *Hoxa5* mutants is not accompanied by change in either cell death or cell proliferation. Cell death and cell proliferation at E14.5 were examined at E14.5 in *Hoxa5* mutants (*n* = 4) and WT littermate controls (*n* = 4). To identify changes in cell death, cleaved Caspase 3 (green), SOX9 (red), and muscle Actin (blue) were analyzed in WT **(A**–**D)**, in both the ventral ICC **(A,B)** and at rib 1 attachment **(C,D)**. Cell death appears minimal in the sternum at both axial levels. Inset in **(A)** indicates positive cleaved Caspase 3 in the dorsal root ganglion. In *Hoxa5* mutants, **(E**–**H)**, cleaved Caspase 3 in the sternum is comparable to that of WT, while an increase in cell death is visible in the pectoral muscles ventral to the sternum **(G,H)**. To identify changes in cell proliferation, expression of PCNA (green), SOX9 (red), and muscle Actin (blue) was examined in WT **(I**–**L)**, in the ventral ICC **(I,J)** and at rib 1 attachment **(K,L)**. At this stage, cell proliferation appears minimal in the sternum at both axial levels, while PCNA levels are higher in developing muscles (pink arrow in **(J)**). In *Hoxa5* mutants, **(M**–**P)**, PCNA expression in the sternum is comparable to that of WT. Scale bar: 200 μM T1, thoracic rib 1.

Supplementary Figure 6 Lateral HOXA5 domain co-expresses EBF3, but not Tenascin. **(A**–**A**‴**)** E14.5 embryo co-labelled for muscle Actin (blue, **A**), HOXA5 (red, **A**′,**A**‴), and Tenascin (green, **A**′,**A**”). HOXA5 and Tenascin are not co-expressed in lateral domain **(A**′**)**. **(B**–**B**‴**)** E14.5 Prx1-Cre-expressing embryo co-labelled for RFP (blue, **B**), HOXA5 (red, **B**′,**B**‴), and EBF3 (green, **B**′,**B**’‘). HOXA5 and EBF3 are co-expressed in lateral domain (**B**′, yellow cells). Orange arrows in **(A,B)** point to HOXA5 lateral domain that does not co-express with SOX9, and corresponds to region indicated in (Supplementary Figure S3C). IC, interclavicular mesenchyme; ICC, intercostoclavicular mesenchyme; PM, pectoral muscle. Scale bar: 200 μM.

## References

[B1] Aubin J. Lemieux M. Moreau J. Lapointe J. Jeannotte L. (2002). Cooperation of Hoxa5 and Pax1 Genes during Formation of the Pectoral Girdle. Developmental Biol. 244 (1), 96–113. 10.1006/dbio.2002.0596 11900462

[B2] Bérubé-Simard F.-A. Jeannotte L. (2014). Hoxa5/Cre Transgenic Mice: Novel Tools for Regional Deletion along the Anterior-Posterior axis. Genesis 52 (2), 149–156. 10.1002/dvg.22733 24307483

[B3] Bickley S. R. B. Logan M. P. O. (2014). Regulatory Modulation of the T-Box Gene Tbx5 Links Development, Evolution, and Adaptation of the Sternum. Proc. Natl. Acad. Sci. U.S.A. 111 (50), 17917–17922. 10.1073/pnas.1409913111 25468972 PMC4273354

[B4] Brent A. E. Braun T. Tabin C. J. (2005). Genetic Analysis of Interactions between the Somitic Muscle, Cartilage and Tendon Cell Lineages during Mouse Development. Development 132 (3), 515–528. 10.1242/dev.01605 15634692

[B5] Buchholtz E. A. Yozgyur Z. M. Feldman A. Weaver A. A. Gaudin T. J. (2020). The Therian Sternum at the Lateral Somitic Frontier: Evolution of a Composite Structure. J. Zool 315, 19–28. 10.1111/jzo.12809

[B6] Burke A. C. Nelson C. E. Morgan B. A. Tabin C. (1995). Hox Genes and the Evolution of Vertebrate Axial Morphology. Development 121 (2), 333–346. 10.1242/dev.121.2.333 7768176

[B7] Burke A. C. Nowicki J. L. (2003). A New View of Patterning Domains in the Vertebrate Mesoderm. Developmental Cel 4 (2), 159–165. 10.1016/S1534-5807(03)00033-9 12586060

[B8] Chen J. M. (1952b). Studies on the Morphogenesis of the Mouse Sternum. II. Experiments on the Origin of the Sternum and its Capacity for Self-Differentiation *In Vitro* . J. Anat. 86 (4), 387–401. Availableat: https://www.ncbi.nlm.nih.gov/pmc/articles/PMC1273691/ . 12999641 PMC1273691

[B9] Chen J. M. (1953). Studies on the Morphogenesis of the Mouse Sternum. III. Experiments on the Closure and Segmentation of the Sternal Bands. J. Anat. 87 (2), 130–149. Availableat: http://www.ncbi.nlm.nih.gov/pubmed/13044725%0Ahttp://www.pubmedcentral.nih.gov/articlerender.fcgi?artid=PMC1244579 . 13044725 PMC1244579

[B10] Chen J. M. (1952a). Studies on the Morphogenesis of the Mouse Sternum. I. Normal Embryonic Development. J. Anat. 86 (4), 373–386. 12999640 PMC1273690

[B11] Chevallier A. (1975). Rôle du me’soderme somitique dans le de’veloppement de la cage thoracique de I'embryon d'oiseau. I. Origine du segment sternal et me’canismes de la diffe’rentiation des côtes. J. Embryol. Exp. Morphol. 33 (2), 291–311. 10.1242/dev.33.2.291 1176848

[B12] Christ B. Huang R. Scaal M. (2007). Amniote Somite Derivatives. Dev. Dyn. 236 (9), 2382–2396. 10.1002/dvdy.21189 17557304

[B13] Compagni A. Logan M. Klein R. Adams R. H. (2003). Control of Skeletal Patterning by EphrinB1-EphB Interactions. Developmental Cel 5 (2), 217–230. 10.1016/S1534-5807(03)00198-9 12919674

[B14] Coulombe Y. Lemieux M. Moreau J. Aubin J. Joksimovic M. Bérubé-Simard F.-A. (2010). Multiple Promoters and Alternative Splicing: Hoxa5 Transcriptional Complexity in the Mouse Embryo. PLoS ONE 5 (5), e10600. 10.1371/journal.pone.0010600 20485555 PMC2868907

[B15] Durland J. L. Sferlazzo M. Logan M. Burke A. C. (2008). Visualizing the Lateral Somitic Frontier in the Prx1Cre Transgenic Mouse. J. Anat. 212 (5), 590–602. 10.1111/j.1469-7580.2008.00879.x 18430087 PMC2409079

[B16] Fell H. B. (1939). The Origin and Developmental Mechanics of the Avian Sternum. Phil. Trans. R. Soc. Lond. B 229 (563), 407–463. 10.1098/RSTB.1939.0002

[B17] Gladstone R. J. Wakeley C. P. (1932). The Morphology of the Sternum and its Relation to the Ribs. J. Anat. 66 (Pt 4), 508–564. Availableat: https://www.ncbi.nlm.nih.gov/pmc/articles/PMC1248911/ . 17104392 PMC1248911

[B18] Holzman M. A. Bergmann J. M. Feldman M. Landry-Truchon K. Jeannotte L. Mansfield J. H. (2018). HOXA5 Protein Expression and Genetic Fate Mapping Show Lineage Restriction in the Developing Musculoskeletal System. Int. J. Dev. Biol. 62 (11–12), 785–796. 10.1387/ijdb.180214jm 30604848 PMC8783609

[B19] Jeannotte L. Gotti F. Landry-Truchon K. (2016). Hoxa5: A Key Player in Development and Disease. J. Dev. Biol. 4 (2). 10.3390/jdb4020013 PMC5831783 29615582

[B20] Jeannotte L. Lemieux M. Charron J. Poirier F. Robertson E. J. (1993). Specification of Axial Identity in the Mouse: Role of the Hoxa-5 (Hox1.3) Gene. Genes Dev. 7 (11), 2085–2096. 10.1101/gad.7.11.2085 7901120

[B21] Jukkola T. Trokovic R. Maj P. Lamberg A. Mankoo B. Pachnis V. (2005). Meox1Cre: A Mouse Line Expressing Cre Recombinase in Somitic Mesoderm. Genesis 43 (3), 148–153. 10.1002/gene.20163 16267823

[B22] Kieny M. Mauger A. Sengel P. (1972). Early Regionalization of the Somitic Mesoderm as Studied by the Development of the Axial Skeleton of the Chick Embryo. Developmental Biol. 28 (1), 142–161. 10.1016/0012-1606(72)90133-9 5041191

[B23] Krumlauf R. (2018). Hox Genes, Clusters and Collinearity. Int. J. Dev. Biol. 62 (11–12), 659–663. 10.1387/ijdb.180330rr 30604835

[B24] Kuriki M. Sato F. Arai H. N. Sogabe M. Kaneko M. Kiyonari H. (2020). Transient and Lineage-Restricted Requirement of Ebf3 for Sternum Ossification. Development (Cambridge) 147 (9). 10.1242/dev.186239 PMC7240299 32398354

[B25] Liem K. F. Bemis W. E. Walker W. F. Grande L. (2001). Functional Anatomy of the Vertebrates: An Evolutionary Perspective. 3rd ed. Philadelphia, PA: Harcourt Brace College Publishers.

[B47] Logan M. Martin J. F. Nagy A. Lobe C. Olson E. N. Tabin C. J. (2002). Expression of Cre Recombinase in the Developing Mouse Limb Bud Driven by a Prxl Enhancer. Genesis 33, 77–80. 10.1002/gene.10092 12112875

[B26] Lufkin T. Mark M. Hart C. P. Dollé P. LeMeur M. Chambon P. (1992). Homeotic Transformation of the Occipital Bones of the Skull by Ectopic Expression of a Homeobox Gene. Nature 359 (6398), 835–841. 10.1038/359835a0 1359423

[B27] Luo Z.-X. Ji Q. Yuan C.-X. (2007). Convergent Dental Adaptations in Pseudo-tribosphenic and Tribosphenic Mammals. Nature 450, 93–97. 10.1038/nature06221 17972884

[B28] Madisen L. Zwingman T. A. Sunkin S. M. Oh S. W. Zariwala H. A. Gu H. (2010). A Robust and High-Throughput Cre Reporting and Characterization System for the Whole Mouse Brain. Nat. Neurosci. 13 (1), 133–140. 10.1038/nn.2467 20023653 PMC2840225

[B29] Mallo M. Wellik D. M. Deschamps J. (2010). Hox Genes and Regional Patterning of the Vertebrate Body Plan. Developmental Biol. 344 (1), 7–15. 10.1016/j.ydbio.2010.04.024 PMC2909379 20435029

[B30] Matsuoka T. Ahlberg P. E. Kessaris N. Iannarelli P. Dennehy U. Richardson W. D. (2005). Neural Crest Origins of the Neck and Shoulder. Nature 436 (7049), 347–355. 10.1038/nature03837 16034409 PMC1352163

[B31] McGlinn E. Holzman M. A. Mansfield J. H. (2019). Detection of Gene and Protein Expression in Mouse Embryos and Tissue Sections. Methods Mol. Biol. 1920. 10.1007/978-1-4939-9009-2_12 30737693

[B32] McIntyre D. C. Rakshit S. Yallowitz A. R. Loken L. Jeannotte L. Capecchi M. R. (2007). Hox Patterning of the Vertebrate Rib Cage. Development 134 (16), 2981–2989. 10.1242/dev.007567 17626057

[B33] Murakami G. Nakamura H. (1991). Somites and the Pattern Formation of Trunk Muscles: A Study in Quail-Chick Chimera. Arch. Histology Cytol. 54 (3), 249–258. 10.1679/aohc.54.249 1954038

[B34] Nowicki J. L. Burke A. C. (2000). Hox Genes and Morphological Identity: Axial versus Lateral Patterning in the Vertebrate Mesoderm. Development 127 (19), 4265–4275. 10.1242/dev.127.19.4265 10976057

[B35] Philippidou P. Walsh C. M. Aubin J. Jeannotte L. Dasen J. S. (2012). Sustained Hox5 Gene Activity Is Required for Respiratory Motor Neuron Development. Nat. Neurosci. 15 (12), 1636–1644. 10.1038/nn.3242 23103965 PMC3676175

[B36] Prummel K. D. Nieuwenhuize S. Mosimann C. (2020). The Lateral Plate Mesoderm, Development 147, 12. 10.1242/dev.175059 PMC7328003 32561665

[B37] Ramírez-Solis R. Zheng H. Whiting J. Krumlauf R. Bradley A. (1993). Hoxb-4 (Hox-2.6) Mutant Mice Show Homeotic Transformation of a Cervical Vertebra and Defects in the Closure of the Sternal Rudiments. Cell 73 (2), 279–294. 10.1016/0092-8674(93)90229-J 8097432

[B38] Rancourt D. E. Tsuzuki T. Capecchi M. R. (1995). Genetic Interaction between Hoxb-5 and Hoxb-6 Is Revealed by Nonallelic Noncomplementation. Genes Dev. 9 (1), 108–122. 10.1101/gad.9.1.108 7828847

[B39] Rodríguez-Vázquez J. F. Verdugo-López S. Garrido J. M. Murakami G. Kim J. H. (20132007). Morphogenesis of the Manubrium of Sternum in Human Embryos: a New Concept. Anat. Rec. 296 (2), 279–289. 10.1002/ar.22623 23165944

[B40] Scaal M. (2021). Development of the Amniote Ventrolateral Body wall. Developmental Dyn. 250 (1), 39–59. 10.1002/dvdy.193 32406962

[B41] Sefton E. M. Kardon G. (2019). Connecting Muscle Development, Birth Defects, and Evolution: An Essential Role for Muscle Connective Tissue. Curr. Top. Developmental Biol. 132, 137–176. 10.1016/bs.ctdb.2018.12.004 PMC6449175 30797508

[B42] Shearman R. M. Burke A. C. (2009). The Lateral Somitic Frontier in Ontogeny and Phylogeny. J. Exp. Zool. 312B (6), 603–612. 10.1002/jez.b.21246 PMC2962407 19021255

[B43] Tabariès S. Lemieux M. Aubin J. Jeannotte L. (2007). Comparative Analysis ofHoxa5 Allelic Series. Genesis 45 (4), 218–228. 10.1002/dvg.20292 17417799

[B44] Tanaka M. Onimaru K. (2012). Acquisition of the Paired Fins: a View from the Sequential Evolution of the Lateral Plate Mesoderm. Evol. Development 14 (5), 412–420. 10.1111/j.1525-142X.2012.00561.x 22947314

[B45] Willey A. (1894). Amphioxus and the Ancestry of the Vertebrates. New York: Macmillan. 10.5962/bhl.title.55924

[B46] Yahya I. Morosan-Puopolo G. Brand-Saberi B. (2020). The CXCR4/SDF-1 Axis in the Development of Facial Expression and Non-somitic Neck Muscles. Front. Cel Dev. Biol. 8, 1674. 10.3389/FCELL.2020.615264 PMC7783292 33415110

