# Supplementary figures and images for: Hoxa5 Activity Across the Lateral Somitic Frontier Regulates Development of the Mouse Sternum
Source: Front Cell Dev Biol. 2022 Apr 26;10:806545. doi: 10.3389/fcell.2022.806545 (PMC9086245; doi:10.3389/fcell.2022.806545)

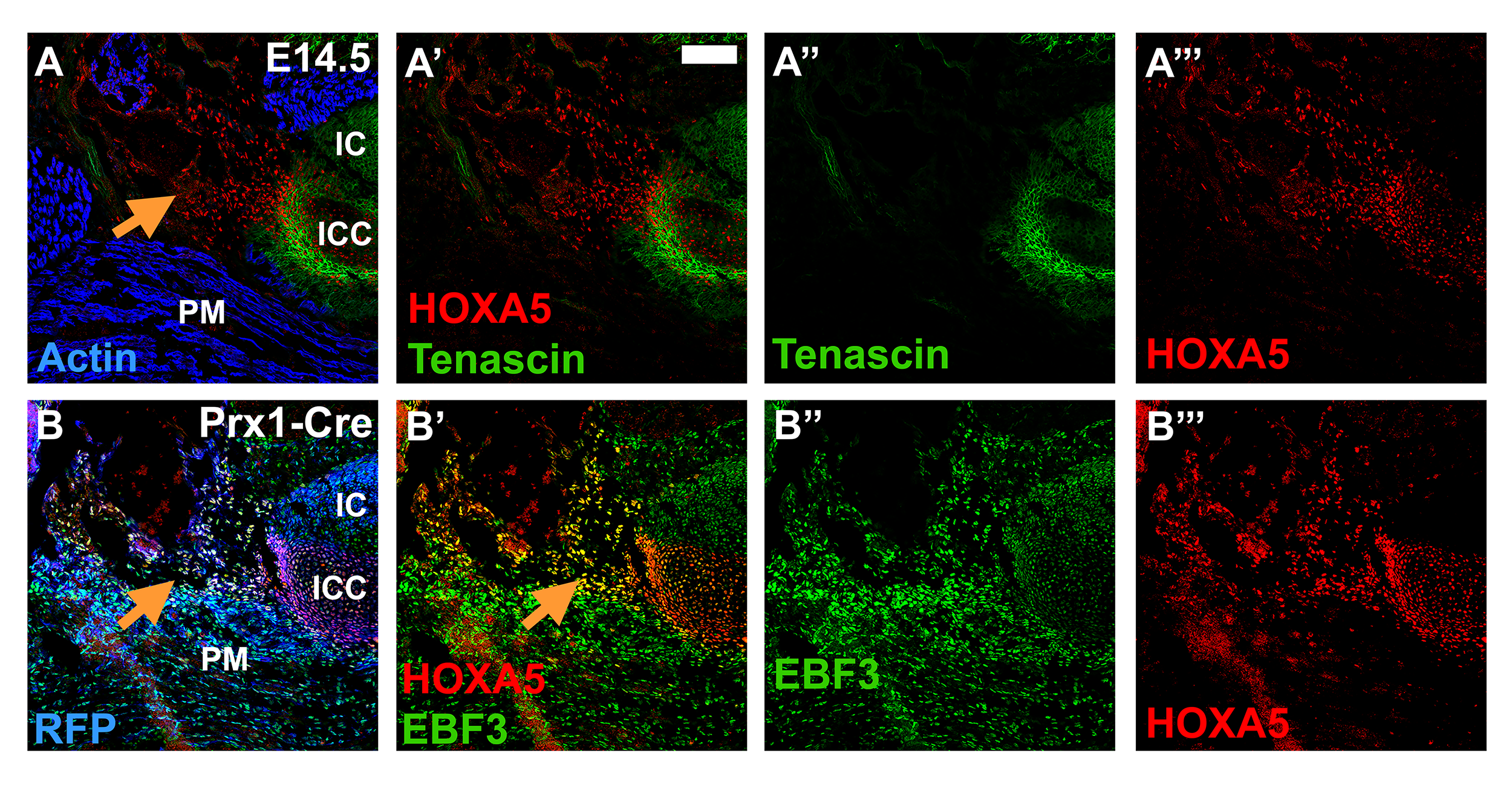

Supplement: Supplementary file 1 [file Image6.tif]

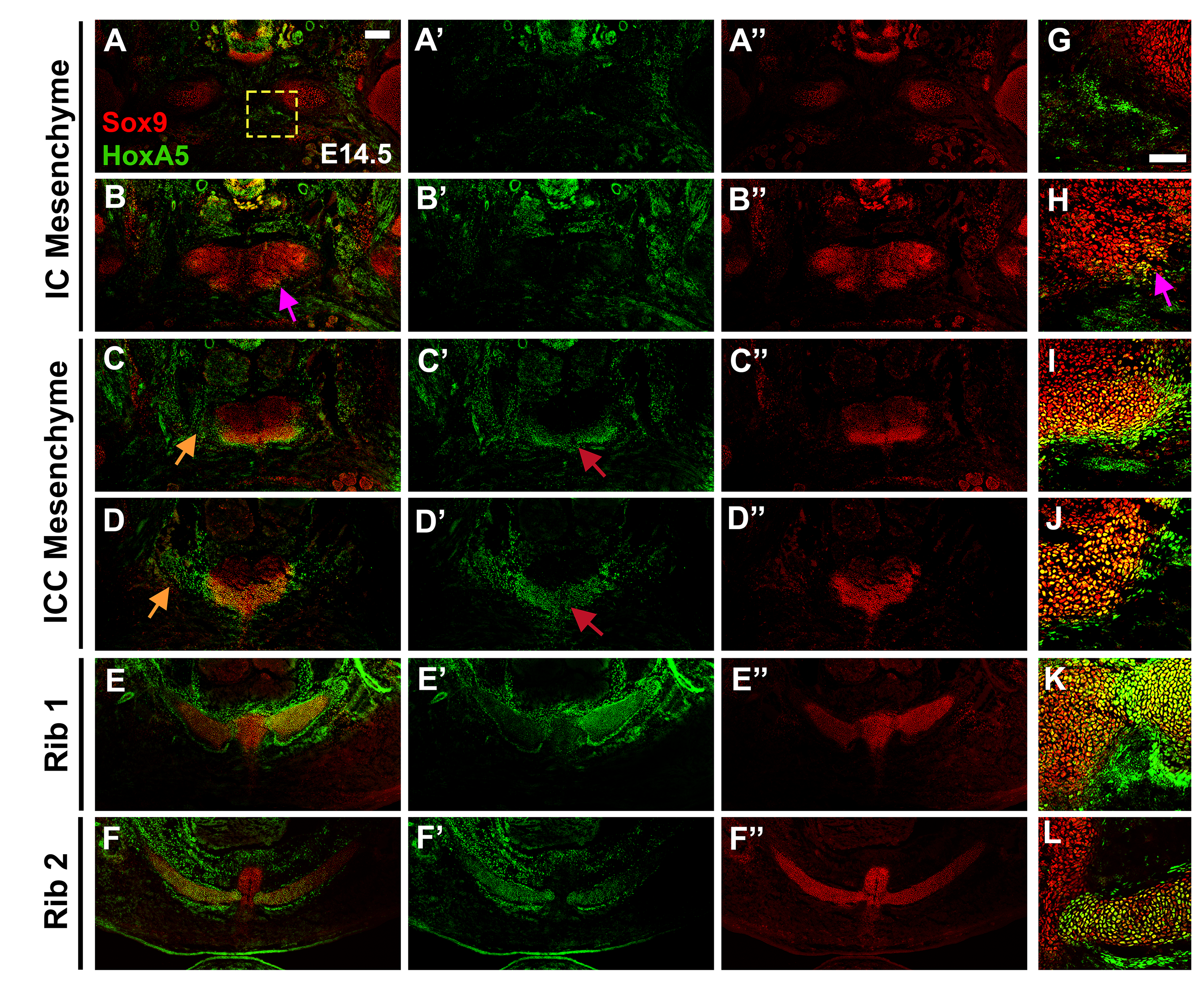

Supplement: Supplementary file 3 [file Image3.TIF]

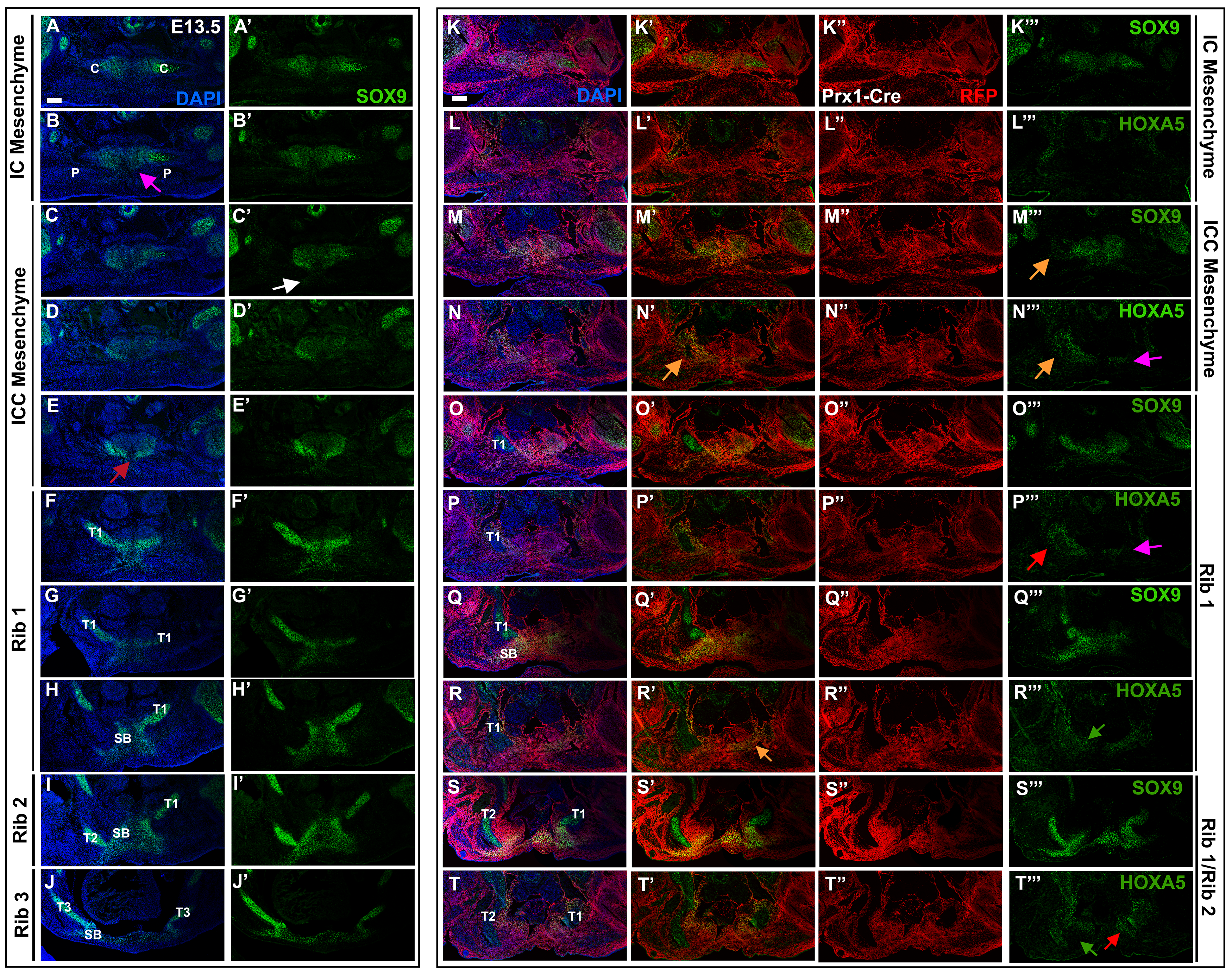

Supplement: Supplementary file 4 [file Image4.TIF]

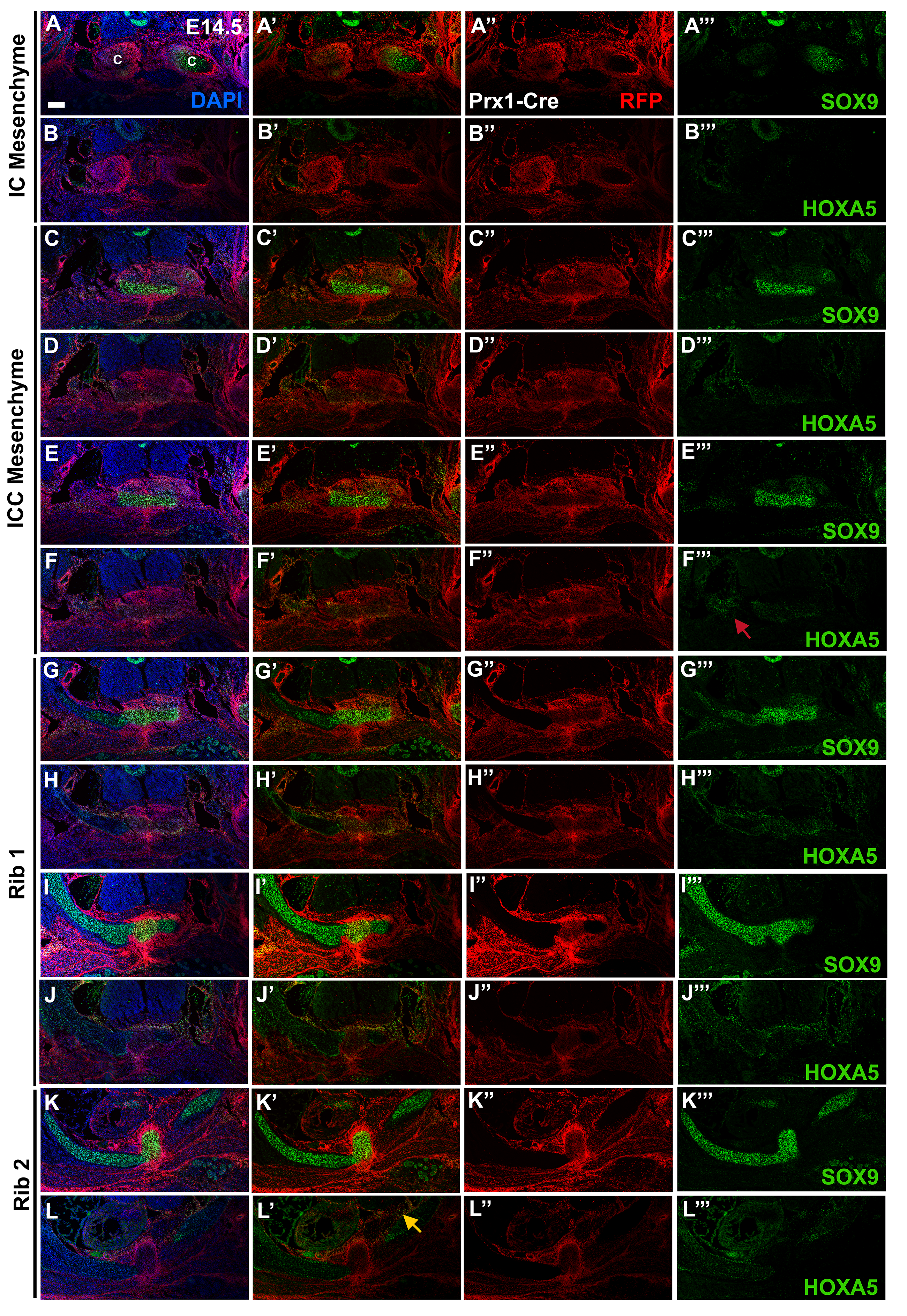

Supplement: Supplementary file 5 [file Image2.TIF]

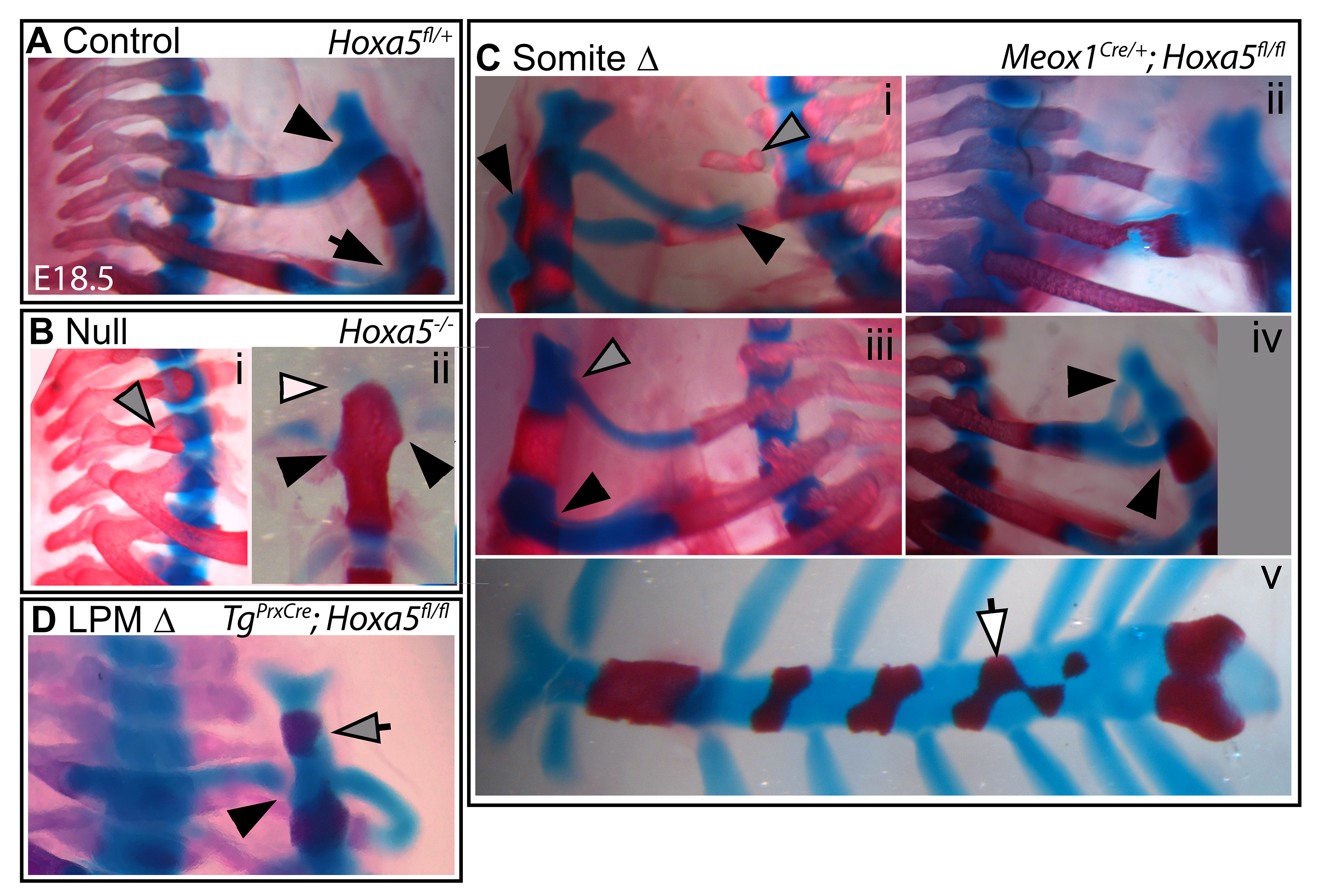

Supplement: Supplementary file 6 [file Image1.TIF]

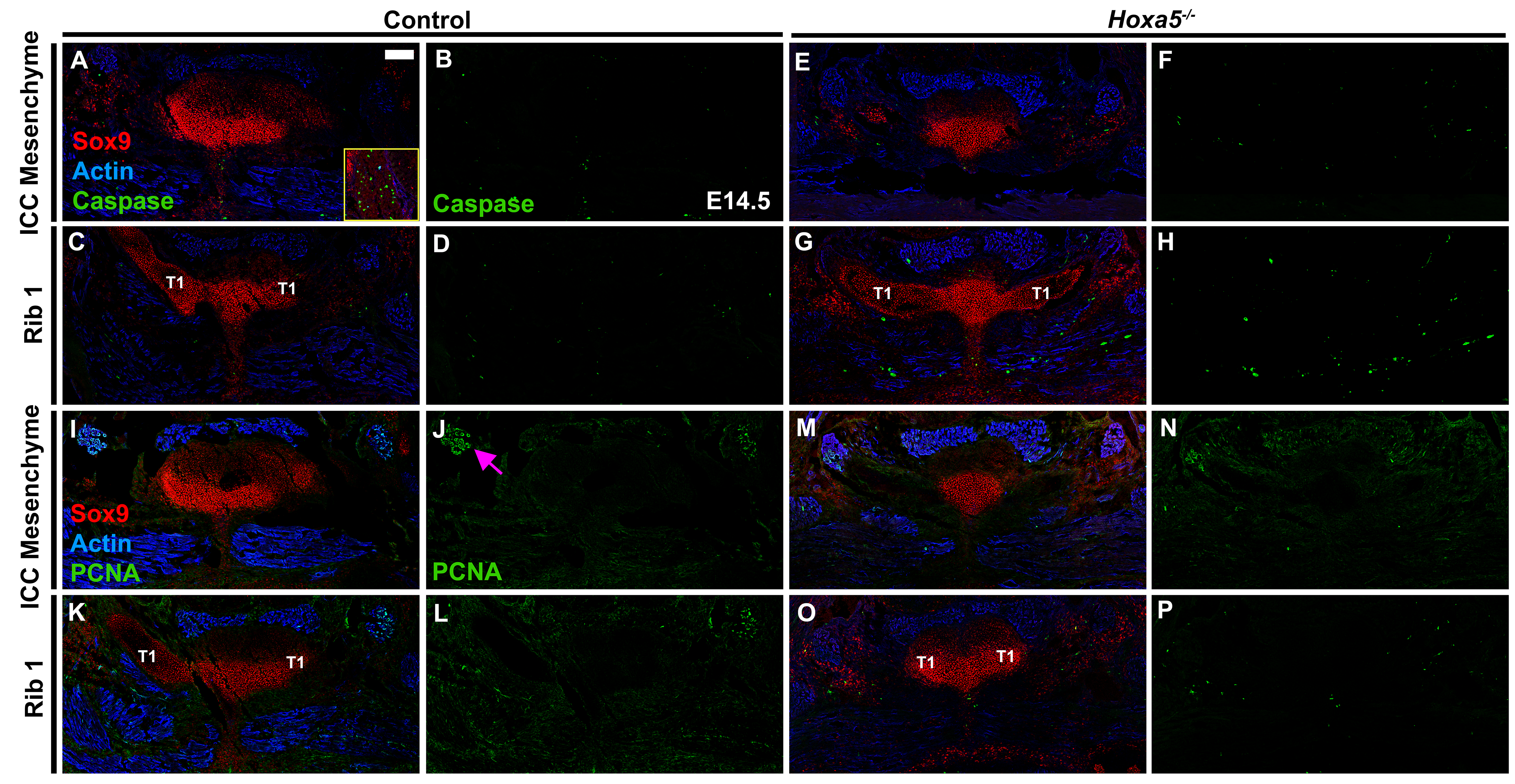

Supplement: Supplementary file 7 [file Image5.TIF]
