# Supplementary material for: Hoxa5 Activity Across the Lateral Somitic Frontier Regulates Development of the Mouse Sternum
Source: Front Cell Dev Biol. 2022 Apr 26;10:806545. doi: 10.3389/fcell.2022.806545 (PMC9086245; doi:10.3389/fcell.2022.806545)
Supplement: Supplementary file 2 [file Table1.pdf]

**Table S1.** Skeletal phenotypes associated with *Hoxa5* loss-of-function are reproduced following conditional deletion in LPM or somites at E18.5. Wild-type phenotypes are listed in bold font and *Hoxa5*-associated phenotypes in regular font. Abbreviations: TA, tuberculum anterior; T1, first thoracic rib; T2, second thoracic rib.

| | | LPM Δ | | | | Somite Δ | | | | Null | | | |
|---|---|---|---|---|---|---|---|---|---|---|---|---|---|
| | | *Hoxa5* $^{fl/+ \text{ or } fl/fl}$ | | *Tg* $^{Prx1\text{-}Cre}$; *Hoxa5* $^{fl/fl}$ | | *Hoxa5* $^{fl/+ \text{ or } fl/fl}$ | | *Meox1* $^{Cre}$; *Hoxa5* $^{fl/fl}$ | | *Hoxa5* $^{+/+}$ | | *Hoxa5* $^{-/-}$ | |
| Structure | Phenotype | # | % | # | % | # | % | # | % | # | % | # | % |
| **C6** | **TA present** | 29 | 85% | 22 | 81% | 19 | 86% | 19 | 37% | 25 | 83% | 4 | 15% |
| | TA absent | 5 | 15% | 5 | 19% | 3 | 14% | 33 | 63% | 5 | 17% | 22 | 85% |
| | | | | | | | | | | | | | |
| **C7** | **Rib absent** | 33 | 97% | 27 | 96% | 22 | 100% | 38 | 73% | 30 | 100% | 19 | 73% |
| | Rib present (all) | 1 | 3% | 1 | 4% | 0 | 0% | 14 | 27% | 0 | 0% | 7 | 27% |
| | Rib fused to T1 | 0 | 0% | 0 | 0% | 0 | 0% | 6 | 12% | 0 | 0% | 0 | 0% |
| | Rib fused to sternum | 0 | 0% | 0 | 0% | 0 | 0% | 1 | 2% | 0 | 0% | 0 | 0% |
| | | | | | | | | | | | | | |
| **T1** | **Dorsal process absent** | 15 | 94% | 14 | 100% | 9 | 90% | 9 | 41% | 12 | 86% | 2 | 17% |
| | Dorsal process present | 1 | 6% | 0 | 0% | 1 | 10% | 13 | 59% | 2 | 14% | 10 | 83% |
| | | | | | | | | | | | | | |
| | | | | | | | | | | | | | |
| **Pre-sternum** | **T1 ribs fused symmetrically and anterior to manubrium ossification** | 17 | 100% | 10 | 71% | 11 | 100% | 19 | 73% | 14 | 93% | 9 | 69% |
| | Asymmetric T1 rib fusion | 0 | 0% | 0 | 0% | 0 | 0% | 7 | 27% | 1 | 7% | 4 | 31% |
| | Extra sternum ossification anterior to T1 | 0 | 0% | 4 | 29% | 0 | 0% | 0 | 0% | 0 | 0% | 0 | 0% |
| | | | | | | | | | | | | | |
| | Ossification flanking T1 attachment | 0 | 0% | 0 | 0% | 0 | 0% | 3 | 3% | 3 | 10% | 12 | 46% |
| | | | | | | | | | | | | | |
| **Meso-sternum** | **Symmetrical sternebrae** | 17 | 100% | 12 | 86% | 10 | 90% | 20 | 77% | 14 | 93% | 13 | 100% |
| | Crankshaft sternum | 0 | 0% | 2 | 14% | 1 | 10% | 6 | 23% | 1 | 7% | 0 | 0% |
| | | | | | | | | | | | | | |
| | Number of Animals (n)* | 17 | | 14 | | 11 | | 26 | | 15 | | 13 | |
